# Oxidative Stress-Related Biomarkers in Inflammatory Bowel Disease: Dual Tools for Remission Assessment and Prediction of Treatment Outcome

**DOI:** 10.3390/antiox14101183

**Published:** 2025-09-28

**Authors:** Armando Tratenšek, Iztok Grabnar, David Drobne, Tomaž Vovk

**Affiliations:** 1 Faculty of Pharmacy, University of Ljubljana, Aškerčeva cesta 7, 1000 Ljubljana, Slovenia; armando.tratensek@ffa.uni-lj.si (A.T.); iztok.grabnar@ffa.uni-lj.si (I.G.); 2 Department of Gastroenterology, University Medical Centre Ljubljana, Japljeva ulica 2, 1000 Ljubljana, Slovenia; david.drobne@kclj.si; 3 Department of Internal Medicine, Faculty of Medicine, University of Ljubljana, Zaloška cesta 7, 1000 Ljubljana, Slovenia

**Keywords:** inflammatory bowel disease, ulcerative colitis, Crohn’s disease, oxidative stress, biomarker, remission

## Abstract

Oxidative stress is increasingly recognized as a significant contributor to the pathogenesis of inflammatory bowel disease (IBD). However, the clinical utility of oxidative stress-related biomarkers for assessing and predicting disease activity remains unclear. This prospective observational study aimed to evaluate the diagnostic and predictive performance of oxidative stress-related biomarkers in distinguishing between active IBD and remission across clinical, biochemical, and endoscopic criteria. A total of 76 patients with IBD were followed across three visits: baseline (biological treatment initiation), post-induction (6–12 weeks), and final follow-up (24–36 weeks). Associations with clinical, biochemical, and endoscopic remission status at the final follow-up were evaluated using correlation matrices, receiver operating characteristic curve analysis, principal component analysis, and logistic regression. Ceruloplasmin, plasma free thiols, gamma-glutamyl transferase, and albumin showed significant diagnostic values for distinguishing active disease from remission, using C-reactive protein (CRP)-based criteria. Serum uric acid, advanced oxidation protein products, gamma-glutamyl transferase, total antioxidant capacity, and ceruloplasmin predicted clinical or CRP-based remission when measured at baseline or post-induction, with predictive value varying by biomarker and the time point. Overall, our findings reinforce the role of oxidative stress in the pathogenesis of IBD and highlight the potential of oxidative stress-related biomarkers to be used as tools for monitoring disease activity and predicting IBD treatment outcomes.

## 1. Introduction

Inflammatory bowel disease (IBD) encompasses two main chronic inflammatory disorders of the gastrointestinal tract: Crohn’s disease (CD) and ulcerative colitis (UC). CD is characterized by transmural inflammation that most commonly affects the ileum and/or colon, but can also occur in any other part of the digestive tract, often in a discontinuous pattern. In contrast, UC is usually confined to the colon and rectum, with inflammation limited to the mucosa. Although the precise etiology remains unclear, IBD is thought to result from an excessive and sustained immune response to intestinal microbiota in genetically susceptible individuals, influenced by various environmental factors. Under normal conditions, the immune response to intestinal microbiota is carefully regulated, maintaining a balance between immune tolerance and inflammatory response [1,2]. In IBD, this balance is disrupted; activated neutrophils and macrophages infiltrate the mucosal layer, triggering inflammation and releasing reactive oxygen and nitrogen species [3]. Reactive species, when regulated by antioxidants, exert beneficial effects in the human body by participating in various cell signaling and redox-dependent metabolic processes. During inflammation, activated phagocytes release large amounts of superoxide anions (O_2_^•−^), hypochlorous acid (HOCl), and hydrogen peroxide (H_2_O_2_) to destroy invading pathogens [3,4]. Enzymatic antioxidants such as superoxide dismutase, catalase, and glutathione peroxidase provide the first line of defense against excessive reactive species in cells, whereas non-enzymatic antioxidants such as albumin, bilirubin, uric acid, thiols, vitamins, and carotenoids constitute the main antioxidants found in plasma [3,4,5]. Chronic inflammation in IBD overwhelms antioxidant defense mechanisms, which become insufficient to neutralize excess reactive species, resulting in oxidative stress and tissue damage [3,6]. This increases intestinal permeability and disrupts gut motility, further contributing to the persistence and even exacerbation of inflammation, highlighting the role of oxidative stress in IBD pathogenesis [3]. Oxidative stress extends beyond the inflamed intestinal mucosa into deeper layers of the intestinal wall. It can also be detected in the systemic circulation through the accumulation of biomarkers of oxidative damage to lipids, proteins, and DNA, as well as by reductions in antioxidants [3,7]. Levels of circulating oxidative stress-related biomarkers differ between active disease and remission and may therefore serve as useful indicators of disease activity [7].

CD and UC are often recognized as progressive disorders, where sustained inflammation can result in irreversible bowel damage. To address this, treatment goals in IBD have evolved significantly over the past decade, shifting from symptom control towards more objective measures such as endoscopic healing [8]. Initial clinical response should be followed by clinical remission. Biochemical remission is initially assessed by normalization of the C-reactive protein (CRP) levels, followed by normalization of fecal calprotectin. Ultimately, long-term treatment goals aim to achieve endoscopic healing and restore the quality of life [8,9]. Therapeutic goals are evolving towards achieving disease clearance and restoring molecular pathways involved in the pathogenesis of IBD, potentially including redox-sensitive signaling pathways [8,10]. Despite evidence linking oxidative stress-related biomarkers with IBD activity, their potential roles in both assessing and predicting clinical, biochemical, and endoscopic remission remain unclear.

This study aimed to evaluate the diagnostic performance of oxidative stress-related biomarkers in distinguishing patients with active IBD from those in remission after 6 months of biological treatment, based on clinical, biochemical, and endoscopic criteria. We also assessed the predictive potential of these biomarkers for identifying patients less likely to respond to therapy and achieve remission using measurements at the start of treatment and at the post-induction visit. Finally, their diagnostic and predictive performances were compared with those of CRP and fecal calprotectin.

## 2. Materials and Methods

### 2.1. Study Design

Our prospective observational study was conducted at a single tertiary referral center. Eligible patients were followed for a minimum of 24 weeks across three predefined visits: Visit 1 (baseline) on the day of biological treatment initiation; Visit 2 (post-induction) between weeks 6 and 12; and Visit 3 (final follow-up) between weeks 24 and 36. Written informed consent was obtained from all participating patients. The study was approved by the National Committee of Medical Ethics (0120-271/2022/4; KME 27 July 2022) and conducted in accordance with the guidelines of the Declaration of Helsinki.

### 2.2. Inclusion Criteria

All consecutive patients aged 18 years or older diagnosed with CD or UC who initiated biological therapy (adalimumab, golimumab, infliximab, ustekinumab, or vedolizumab) between June 2023 and February 2024 were considered for inclusion. Therapy was initiated by a multidisciplinary team based on clinical, biochemical, or endoscopic evidence of active disease at baseline.

### 2.3. Exclusion Criteria

Patients with comorbidities that could potentially influence oxidative stress–related or inflammatory parameters were excluded. These included neurodegenerative diseases (such as Alzheimer’s disease, Parkinson’s disease, multiple sclerosis, schizophrenia), cerebrovascular diseases, active malignancies, lipid metabolism disorders, and endocrine disorders (e.g., diabetes mellitus, hyperthyroidism, hypothyroidism). Patients with impaired liver or kidney function, other chronic inflammatory conditions, alcohol dependence, obesity, or cachexia were also excluded. The use of antioxidant supplements was an exclusion criterion as well. Patients who discontinued treatment or switched to non-biological therapies were included in the analysis up to that point but excluded from further follow-up.

### 2.4. Biomarker Measurements

Blood and stool samples were prospectively collected at Visit 1, Visit 2, and Visit 3. Routine laboratory parameters, including complete blood count, electrolytes, liver function tests, total iron-binding capacity (TIBC), C-reactive protein (CRP), serum uric acid (SUA), and ceruloplasmin were immediately measured using standard automated clinical laboratory assays. Plasma samples were then stored at −80 °C until batch analysis of advanced oxidation protein products (AOPP), plasma free thiols (R-SH), malondialdehyde (MDA), and total antioxidant capacity (TAC) were performed.

AOPP and R-SH were both measured spectrophotometrically using a Tecan Spark multimode microplate reader (Tecan Group Ltd., Männedorf, Switzerland) at 340 nm and 412 nm, following the methods described by Taylor et al. [11] and Taylan et al. [12], respectively.

Plasma MDA levels were quantified using a derivatization approach with 2,4-dinitrophenylhydrazine (DNPH, Sigma-Aldrich, Steinheim, Germany) [13]. Following derivatization, the samples were analyzed using high-performance liquid chromatography on an Agilent 1100 Series system (Agilent Technologies, Santa Clara, CA, USA). Chromatographic separation was achieved with an Agilent Eclipse XBD-C18 column (5 µm, 150 mm × 4.6 mm, Agilent Technologies, Santa Clara, CA, USA). A gradient elution method was applied, utilizing a mobile phase consisting of 0.2% acetic acid (Merck, Darmstadt, Germany) in MilliQ water (Millipore Corp., Billerica, MA, USA) (mobile phase A) and 0.2% acetic acid in acetonitrile (Sigma-Aldrich, Steinheim, Germany) (mobile phase B). The flow rate was set at 1.1 mL/min and the following gradient was employed (% of mobile phase B): 25.0, 38.0, and 25.0 at the corresponding time points: 0, 13.0, and 13.1 min, respectively. The MDA derivative was detected at 310 nm using an ultraviolet detector.

TAC plasma levels were measured using a commercially available Antioxidant Assay Kit (Cat. #709001; Cayman Chemical, Ann Arbor, MI, USA). This method relies on the ability of both aqueous and lipid-soluble antioxidants to inhibit the oxidation of 2,2′-azino-bis(3-ethylbenzothiazoline-6-sulfonic acid) (ABTS) to its radical cation form (ABTS^+^). The resulting absorbance was measured spectrophotometrically at 750 nm using a Tecan Spark multimode microplate reader (Tecan Group Ltd., Männedorf, Switzerland).

Fecal samples of the first morning bowel movement were collected at the same time points for calprotectin measurements using the Calprest ELISA assay (Eurospital, Trieste, Italy) with a measurement range of 27–2700 mg/kg.

### 2.5. Disease Activity Assessment

Disease activity was evaluated at each visit using clinical and biochemical criteria. Additionally, endoscopic criteria were used when available.

Clinical activity. A two-item Patient-Reported Outcome (PRO-2) was used to assess clinical disease activity, with higher scores indicating more active disease. In CD, disease activity was evaluated using the Abdominal Pain Score (APS) and the number of daily liquid stools. APS was graded on a 0–3 scale: 0—none, 1—mild, 2—moderate, and 3—severe. In UC, disease activity was assessed using the Rectal Bleeding Score (RBS) and the Stool Frequency Score (SFS). RBS was graded on a 0–3 scale: 0—no blood seen, 1—streaks of blood with stool less than half of the time, 2—obvious blood with stool most of the time, and 3—blood alone passes. SFS was also graded on a 0–3 scale based on deviations from the patient’s normal stool frequency: 0—normal number of stools for the patient, 1—1 to 2 stools per day more than normal, 2—3 to 4 stools per day more than normal, and 3—≥5 stools per day more than normal.

Biochemical activity. CRP [mg/L] and fecal calprotectin [mg/kg] measurements were used to assess the biochemical activity of the disease, with higher values indicating greater disease activity.

Endoscopic activity. The Simple Endoscopic Score for CD (SES-CD) [14] and the Mayo endoscopic subscore [15] for UC were used to assess endoscopic disease activity. The SES-CD evaluates the rectum, left colon, transverse colon, right colon, and ileum, grading four variables in each segment (size of ulcers, extent of ulcerated surface, extent of affected surface, and presence/type of narrowing) on a 0–3 scale. A score of 0 indicates no lesions, whereas a score of 3 reflects the most severe findings (e.g., very large ulcers, >30% ulcerated surface, >75% affected surface, or non-passable stenosis). The total SES-CD is calculated as the sum across all segments (range: 0–60 points). The Mayo endoscopic subscore is graded on a 0–3 scale: 0—normal mucosa, 1—mild activity (erythema, lack of vascular pattern, mild friability), 2—moderate activity (marked erythema, absent vascular pattern, friability, erosions), and 3—severe activity (spontaneous bleeding, ulceration).

### 2.6. Disease Remission Criteria

Remission was defined according to established clinical, biochemical, and endoscopic criteria, with disease activity scores and scoring systems detailed in Section 2.5. Specific cutoff values used to define remission are provided below.

Clinical remission was defined using PRO-2 criteria: APS ≤ 1 and a daily liquid stool frequency ≤ 1.5 in CD [16], and RBS = 0 and SFS ≤ 1 in UC [17].

Biochemical remission based on CRP was defined as CRP < 5 mg/L, corresponding to the lower limit of quantification in our laboratory. Biochemical remission based on fecal calprotectin was defined as calprotectin < 100 mg/kg. The cutoff was selected based on test characteristics reported in meta-analyses [18], with particular emphasis on studies that used the same assay as our center [19]. The same cut off was used in our previous trial and was applied here for consistency [20].

Endoscopic remission was defined as an SES-CD score < 3 without mucosal ulceration in CD, and a Mayo endoscopic subscore of 0 in UC [9].

### 2.7. Statistical Analysis

Descriptive statistics were presented as frequencies and percentages for categorical variables, and as medians with interquartile ranges (IQR) for continuous variables.

Hierarchical clustering of oxidative stress-related biomarkers was performed to explore the underlying redox-related patterns at baseline, using only individuals with complete biomarker data. Biomarker values were standardized as Z-scores, and clustering was conducted using complete linkage and Euclidean distance. Additionally, patients were annotated with clinical and demographic factors (drug, biologic-naïve status, smoking status, sex, and IBD subtype) to evaluate potential confounding effects.

Spearman’s rank-order correlation coefficient was used to evaluate associations between continuous variables. Next, a correlation matrix was constructed to visualize relationships among oxidative stress-related biomarkers and disease activity at Visit 3.

The diagnostic performance of individual oxidative stress-related biomarkers in distinguishing between active IBD and remission at the final follow-up visit was evaluated using receiver operating characteristic (ROC) curve analysis. The area under the ROC curve (AUC), along with its 95% confidence interval (CI), was calculated. Optimal thresholds were determined using Youden’s J statistic [21]. According to conventional interpretation, an AUC of 0.5 indicates no discriminatory ability, 0.6 ≤ AUC < 0.7 indicates poor discrimination, 0.7 ≤ AUC < 0.8 is considered acceptable, 0.8 ≤ AUC < 0.9 is considered excellent, and values ≥ 0.9 represent outstanding diagnostic performance [22,23]. ROC curves were compared using DeLong’s method [24]. The Mann–Whitney U test (two-sided) was used to compare oxidative stress-related biomarker levels between patients who achieved and those who did not achieve the remission endpoint. Raw *p*-values were adjusted for multiple comparisons using the Benjamini–Hochberg procedure to control the false discovery rate at 0.05, thereby reducing the risk of false positives and ensuring more robust identification of significant associations [25].

The predictive performance of oxidative stress–related biomarkers in distinguishing between active IBD and remission at the final follow-up was evaluated using ROC curve analysis, based on biomarker data collected at baseline and at Visit 2 (post-induction). ROC curve analysis, AUC interpretation and Mann–Whitney U test were performed as described in the diagnostic performance evaluation.

Principal component analysis (PCA) was conducted using the princomp() function in R to reduce dimensionality and identify combinations of oxidative stress–related biomarkers that best distinguish between active IBD and remission at Visit 3. Before analysis, biomarker values were standardized as Z-scores using the scale() function. To minimize redundancy, for biomarker pairs with an absolute Spearman’s correlation coefficient > 0.9, only one biomarker was retained. Only patients with complete data for all biomarkers were included in the analysis. Principal components (PCs) were selected based on eigenvalues greater than 1 (Kaiser rule) [26], visual inspection of scree plots, and cumulative variance explained.

Univariable logistic regression analysis was performed to evaluate the association between each PCA-derived PC and the remission outcomes. PC scores were included as predictors in separate models for each remission criterion. Odds ratios (ORs) with 95% CIs were reported. *p*-values and 95% CIs were obtained using the log-likelihood ratio test.

Multivariable logistic regression was used to assess the diagnostic performance of various combinations of biomarkers in distinguishing active from inactive IBD at the final follow-up. The biomarker with the highest AUC from individual ROC analysis was combined with biomarkers from PCA-derived PCs associated with remission. An absolute loading threshold of >0.4 was applied to select biomarkers contributing to these components. Predicted probabilities were then used to generate ROC curves and calculate AUC, following the same approach and interpretation as described in the diagnostic performance evaluation.

Patients were included in each analysis only if they had complete data for the relevant biomarkers and outcomes, with no imputation for missing values. All statistical analyses were performed using R software (version 4.4.1, R Development Core Team, Vienna, Austria) within RStudio (version 2024.09.0, RStudio, Boston, MA, USA). The “pheatmap”, “ggcorrplot”, and “pROC” packages were used to create the heatmap, generate the correlation matrix, and conduct ROC analyses, respectively. A *p*-value < 0.05 was considered statistically significant.

## 3. Results

### 3.1. Patient Baseline Characteristics

A total of 76 patients with IBD (40 with CD and 36 with UC) were enrolled in the study. The median age was 46 years (IQR 31–58), and 35 patients were classified as female based on sex recorded in medical records. The median disease duration was 6 years (IQR 3–16). The median weight and height were 72 kg (IQR 62–84) and 172 cm (IQR 165–179), respectively. Smoking status included 43 patients who had never smoked, 21 former smokers, and 12 active smokers. Prior exposure to biological therapy was reported in 38 patients. At baseline, treatment was initiated with ustekinumab (*n* = 27), vedolizumab (*n* = 27), infliximab (*n* = 13), adalimumab (*n* = 8), and golimumab (*n* = 1). Concomitant therapies included oral 5-aminosalicylic acid (*n* = 12), oral locally acting corticosteroids (*n* = 9), enteral nutrition (*n* = 8), oral systemic corticosteroids (*n* = 7), topical 5-aminosalicylic acid (*n* = 5), azathioprine (*n* = 4), topical corticosteroids (*n* = 4), upadacitinib (*n* = 2), and filgotinib (*n* = 1).

**Figure 1:**
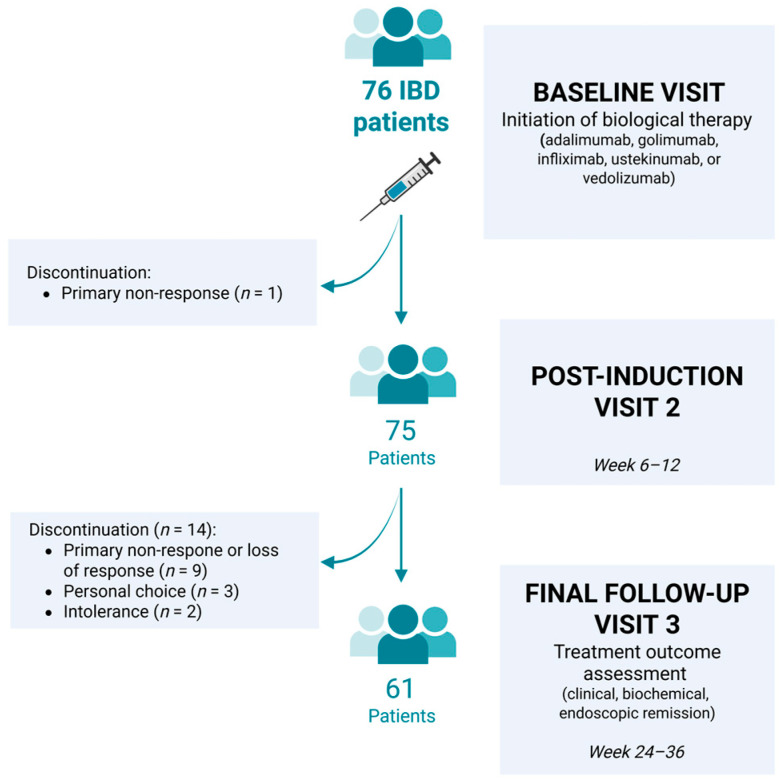
Study workflow and patient flow. Seventy-six consecutive patients with inflammatory bowel disease (IBD) initiated biological therapy (adalimumab, golimumab, infliximab, ustekinumab, or vedolizumab) at baseline (Visit 1). One patient discontinued due to primary non-response before Visit 2 (post-induction, week 6–12). Fourteen additional patients discontinued before the final follow-up (Visit 3, week 24–36) due to primary non-response or loss of response (*n* = 9), personal choice (*n* = 3), or intolerance (*n* = 2). Sixty-one patients completed the study and were assessed for clinical, biochemical, and endoscopic remission.

Of the 76 patients enrolled, 1 discontinued after Visit 1 due to primary non-response and a switch to a non-eligible therapy. After Visit 2, an additional 14 patients discontinued the study for the following reasons: primary non-response or loss of response (*n* = 9), personal choice (*n* = 3), and intolerance (*n* = 2). Complete follow-up through Visit 3 was available for 61 patients (32 with CD and 29 with UC), of whom 31 were female. The study design is outlined in Figure 1.

### 3.2. Profiling and Clustering of Oxidative Stress-Related Biomarkers

A total of 15 oxidative stress-related biomarkers were profiled at each visit, and clustering analysis was performed on baseline data to explore the corresponding redox-related patterns. The biomarkers assessed included albumin, AOPP, ceruloplasmin, direct bilirubin, ferritin, gamma-glutamyl transferase (GGT), hemoglobin, iron, MDA, R-SH, SUA, TAC, total bilirubin, TIBC, and unsaturated iron-binding capacity (UIBC). In addition, CRP and fecal calprotectin were included as key markers indicative of inflammation. Median values, IQR, and the number of patients with available measurements for each visit are provided in Table 1.

**Figure 2:**
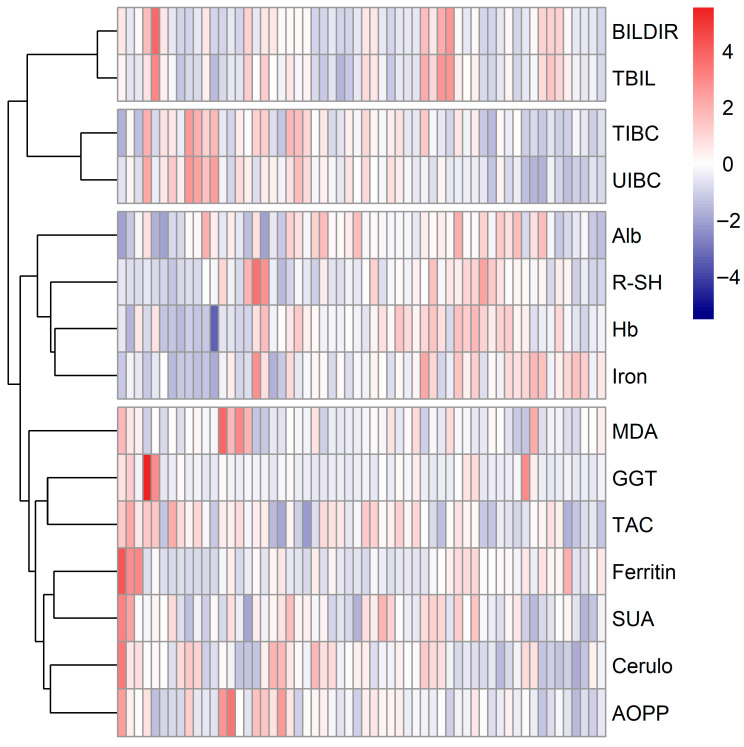
Heatmap showing hierarchical clustering of 15 oxidative stress-related biomarkers in patients with IBD at baseline. Patients with complete biomarker data were included (*n* = 58), and biomarker values were Z-score standardized. A centered color scale was applied with red indicating above-average and blue indicating below-average biomarker levels across the cohort. Rows represent individual biomarkers, and columns represent individual patients. Abbreviations: Alb, albumin; AOPP, advanced oxidation protein products; BILDIR, bilirubin (direct); Cerulo, ceruloplasmin; GGT, gamma-glutamyl transferase; Hb, hemoglobin; MDA, malondialdehyde; R-SH, plasma free thiols; SUA, serum uric acid; TAC, total antioxidant capacity; TBIL, total bilirubin; TIBC, total iron-binding capacity; UIBC, unsaturated iron-binding capacity.

Hierarchical clustering of baseline measurements was performed, as this time point provided the most complete dataset, with all 15 oxidative stress–related biomarkers available for 58 individuals. Standardized values (Z-scores) were visualized in a heatmap. We have observed, that several biomarkers with similar biological roles clustered together. Antioxidant-related biomarkers and negative acute phase reactants (albumin, R-SH, iron) grouped closely, while markers of oxidative damage (AOPP, MDA) clustered together with positive acute-phase reactants (ferritin, ceruloplasmin). Further details on the underlying redox-related patterns at baseline are outlined in Figure 2. Additionally, patients annotated with clinical and demographic factors (drug, biologic-naïve status, smoking status, sex, and IBD subtype) showed no clear grouping by these variables (Figure S1).

### 3.3. Clinical Outcomes and Associations with Oxidative Stress-Related Biomarkers

**Figure 3:**
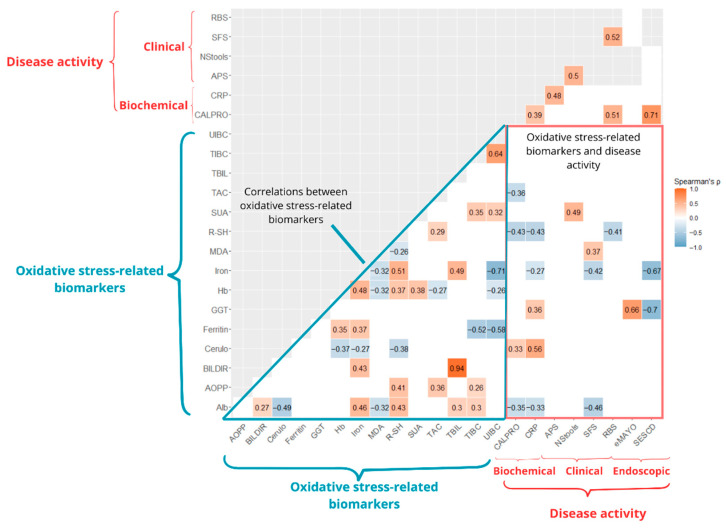
Correlation matrix of oxidative stress-related biomarkers and disease activity parameters at Visit 3. Sample sizes varied from 7 to 61, depending on the variables compared. The matrix presents pairwise Spearman correlation coefficients (ρ). Only statistically significant correlations (*p* < 0.05) are shown with their respective ρ values. Positive correlations are shown in red and negative correlations in blue; color intensity indicates the strength of the correlation. Alb, albumin; AOPP, advanced oxidation protein products; APS, abdominal pain score; BILDIR, direct bilirubin; CALPRO, fecal calprotectin; Cerulo, ceruloplasmin; CRP, C-reactive protein; GGT, gamma-glutamyl transferase; Hb, hemoglobin; Iron, serum iron; MDA, malondialdehyde; NStools, number of daily liquid stools; RBS, rectal bleeding score; R-SH, plasma free thiols; SFS, stool frequency score; SUA, serum uric acid; TAC, total antioxidant capacity; TBIL, total bilirubin; TIBC, total iron-binding capacity; UIBC, unsaturated iron-binding capacity.

The main treatment goal was to achieve remission at the final follow-up (Visit 3). Therefore, the relationships between oxidative stress–related biomarkers and disease activity parameters were evaluated at this time point (Figure 3). Remission rates across all visits are provided in Table 2, based on per-protocol analysis of patients with complete data at each visit.

Several significant associations were observed both among oxidative stress-related biomarkers and between these biomarkers and conventional indicators of disease activity at Visit 3. CRP showed a positive correlation with ceruloplasmin (ρ = 0.56, *p* < 0.001) and GGT (ρ = 0.36, *p* = 0.005), and a negative correlation with R-SH (ρ = −0.43, *p* < 0.001), albumin (ρ = −0.33, *p* = 0.01), and iron (ρ = −0.27, *p* = 0.034). Fecal calprotectin was positively correlated with ceruloplasmin (ρ = 0.33, *p* = 0.031) and negatively correlated with R-SH (ρ = −0.43, *p* = 0.003), albumin (ρ = −0.35, *p* = 0.021), and TAC (ρ = −0.36, *p* = 0.013). RBS was negatively correlated with R-SH (ρ = −0.41, *p* = 0.028), while SFS was negatively correlated with albumin (ρ = −0.46, *p* = 0.014) and iron (ρ = −0.42, *p* = 0.023), and positively correlated with MDA (ρ = 0.37, *p* = 0.049). Number of stools was positively correlated with SUA (ρ = 0.49, *p* = 0.010). SES-CD was negatively correlated with GGT (ρ = −0.70, *p* = 0.035) and iron (ρ = −0.67, *p* = 0.047), while the endoscopic Mayo score showed a positive correlation with GGT (ρ = 0.66, *p* = 0.007). Further details are provided in Figure 3.

### 3.4. Diagnostic Performance of Oxidative Stress-Related Biomarkers for Remission Assessment at the Final Follow-Up

**Figure 4:**
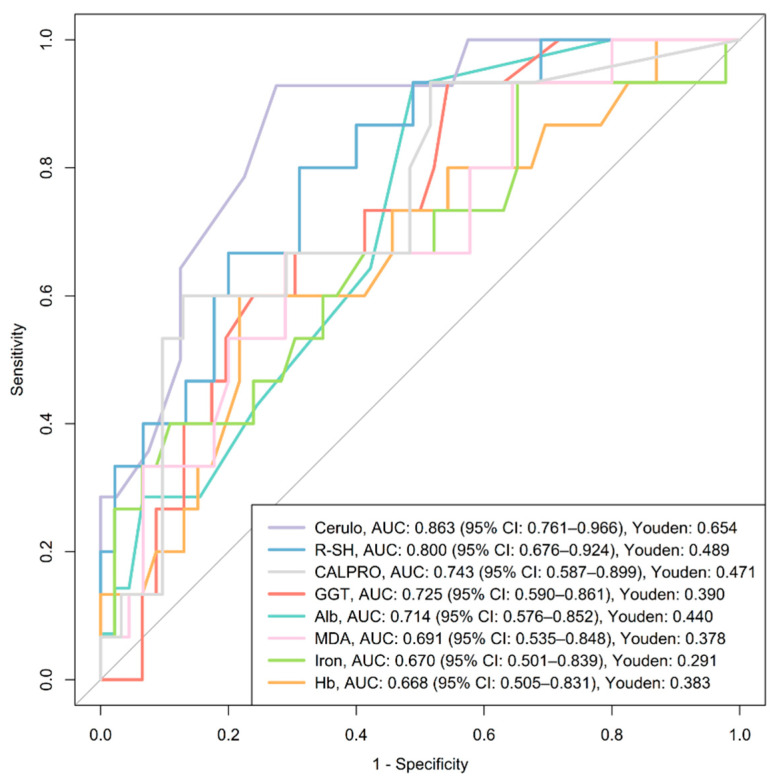
ROC curves evaluating the diagnostic performance of oxidative stress-related biomarkers in distinguishing between active disease and remission based on CRP criteria at the final visit. Only biomarkers with the lower bound of the AUC 95% CI above 0.5 are shown. After adjustment for multiple comparisons (Benjamini–Hochberg), ceruloplasmin, R-SH, calprotectin, GGT, and albumin remained significant. Calprotectin was included for comparison. Abbreviations: Alb, albumin; AUC, area under the receiver operating curve; Cerulo, ceruloplasmin; CI, confidence interval; CALPRO, fecal calprotectin; GGT, gamma-glutamyl transferase; Hb, hemoglobin; MDA, malondialdehyde; R-SH, plasma free thiols; Youden, Youden Index.

ROC curve analysis was performed to assess the ability of oxidative stress–related biomarkers to distinguish between active IBD and remission based on clinical, CRP, calprotectin, and endoscopic criteria at the final follow-up (Visit 3), reflecting outcomes after approximately 6 months of therapy with biologics. Up to 61 patients were included for clinical and CRP-based remission, 46 for calprotectin-based remission, and 32 for endoscopic remission, depending on the availability of paired biomarker and outcome data (see Table 1, Table 2 and Table 3). After adjusting *p*-values for multiple comparisons (Benjamini–Hochberg), only the CRP-based remission analysis yielded significant findings. Full diagnostic metrics for CRP remission are shown in Table 3. Figure 4 illustrates ROC curves for all biomarkers with a lower 95% CI of AUC above 0.5, with ceruloplasmin, R-SH, calprotectin, GGT, and albumin remaining significant after adjustment (Benjamini–Hochberg). Results for other remission types are provided in Supplementary Materials, Table S1.

Ceruloplasmin showed the highest diagnostic accuracy for CRP-based biochemical remission (AUC 0.863), with elevated levels in active disease (median: 0.30 g/L, IQR: 0.28–0.34) versus remission (median: 0.24 g/L, IQR: 0.21–0.27; *p* < 0.001). R-SH demonstrated excellent diagnostic accuracy for CRP-based biochemical remission (AUC 0.800), with lower levels in active disease (median: 420 µmol/L, IQR: 373–464) compared to remission (median: 512 µmol/L, IQR: 447–578; *p* = 0.002). Albumin showed a similar pattern (AUC 0.714), with concentrations reduced in active disease (median: 44 g/L, IQR: 40.5–45) compared to remission (median: 46 g/L, IQR: 44–46; *p* = 0.048). GGT demonstrated acceptable diagnostic accuracy for CRP-based biochemical remission (AUC 0.725), with higher levels in active disease (median: 0.49 µkat/L, IQR: 0.32–0.80) compared to remission (median: 0.30 µkat/L, IQR: 0.22–0.45; *p* = 0.038) (Table 3A, Figure 4).

In subgroup analyses, no biomarkers remained significant for CD after adjustment. In UC, ceruloplasmin showed the best performance (AUC 0.912, 95% CI 0.774–1.000), with higher levels in active disease (median 0.31 g/L, IQR 0.28–0.35; *n* = 9) versus remission (median 0.25 g/L, IQR 0.22–0.26; *n* = 17; *p* = 0.011).

### 3.5. Predictive Performance of Oxidative Stress-Related Biomarkers for Remission Assessment at the Final Follow-Up

Biomarkers measured at the post-induction (Visit 2) and at baseline were evaluated for their ability to predict treatment outcomes at the final follow-up using ROC curve analysis. Outcomes included clinical remission (*n* = 61), biochemical remission based on CRP (*n* = 61), fecal calprotectin (*n* = 46), and endoscopic remission (*n* = 32). Sample sizes varied by outcome and data availability. None of the oxidative stress–related biomarkers showed significant predictive ability for calprotectin-based or endoscopic remission after adjustment for multiple comparisons, regardless of being measured at the post-induction visit or at the baseline. Overall, the results below focus on clinical and CRP-based remission. Full diagnostic metrics for CRP-based remission are provided in Table 3B,C, while metrics for clinical, calprotectin-based, and endoscopic remission are presented in Supplementary Material, Table S2 (post-induction Visit 2) and Table S3 (baseline). Subgroup analyses for CD and UC were performed throughout, but due to limited sample sizes most biomarkers lost significance after adjustment for multiple comparisons unless otherwise noted.

#### 3.5.1. Prediction Based on Post-Induction Visit 2 Biomarkers

##### Clinical Remission

**Figure 5:**
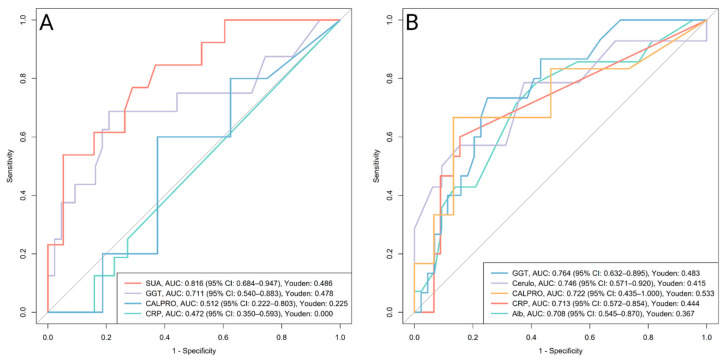
ROC curves evaluating the predictive performance of oxidative stress-related biomarkers at Visit 2 in distinguishing between active disease and remission at Visit 3 based on (**A**) clinical (*n* = 61) and (**B**) biochemical (CRP; *n* = 61) criteria. Only biomarkers with a lower 95% CI above 0.5 are shown. After adjustment for multiple comparisons, only SUA (**A**) and GGT, ceruloplasmin, and CRP (**B**) remained significant. CRP and calprotectin are shown for comparison. Abbreviations: Alb, albumin; AUC, area under the receiver operating curve; Cerulo, ceruloplasmin; CI, confidence interval; CALPRO, fecal calprotectin; CRP, C-reactive protein; GGT, gamma-glutamyl transferase; IBD, inflammatory bowel disease; MDA, malondialdehyde; R-SH, plasma free thiols; SUA, serum uric acid; Youden, Youden Index.

SUA measured at Visit 2 demonstrated the strongest predictive performance for clinical activity at Visit 3 (AUC 0.816, 95% CI 0.684–0.947). SUA was the only biomarker that remained statistically significant after adjustment for multiple comparisons, with higher levels in patients who later had clinically active disease (median: 432 µmol/L, IQR: 310–462) compared to those in remission (median: 273 µmol/L, IQR: 234–312; *p* = 0.013). In contrast, CRP and calprotectin were not significant predictors of clinical remission (Figure 5A, Supplementary Material, Table S2).

##### Biochemical Remission Based on CRP

At Visit 2, both GGT (AUC 0.764) and ceruloplasmin (AUC 0.746) showed stronger predictive performance for CRP-based remission outcome at follow-up than CRP or calprotectin and remained significant after adjustment for multiple comparisons (Figure 5B, Table 3B). Levels were higher in patients who later had active disease, with GGT at 0.44 µkat/L (IQR: 0.37–0.70) versus 0.29 µkat/L (IQR: 0.24–0.39; *p* = 0.021) and ceruloplasmin at 0.32 g/L (IQR: 0.27–0.36) versus 0.26 g/L (IQR: 0.24–0.28; *p* = 0.049) in remission.

#### 3.5.2. Prediction Based on Baseline Biomarkers (Visit 1)

##### Clinical Remission

**Figure 6:**
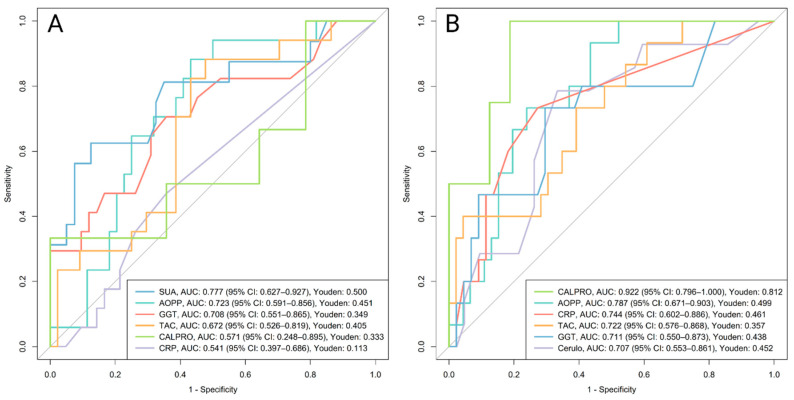
ROC curves evaluating the predictive performance of baseline oxidative stress-related biomarkers in distinguishing between active disease and remission at Visit 3 based on (**A**) clinical (*n* = 61) and (**B**) biochemical (CRP; *n* = 61) criteria. Only biomarkers with a lower 95% CI above 0.5 are shown. CRP and calprotectin are included for comparison. After adjustment for multiple comparisons, only SUA (**A**) and AOPP, TAC, calprotectin, and CRP (**B**) remained significant. Abbreviations: AOPP, advanced oxidation protein products; AUC, area under the receiver operating curve; Cerulo, ceruloplasmin; CI, confidence interval; CALPRO, fecal calprotectin; CRP, C-reactive protein; GGT, gamma-glutamyl transferase; IBD, inflammatory bowel disease; SUA, serum uric acid; TAC, total antioxidant capacity; Youden, Youden Index.

After adjustment for multiple comparisons, baseline SUA remained the only significant predictor of clinical activity at Visit 3, with an AUC of 0.777 (95% CI 0.627–0.927). Baseline levels were higher in patients who later had active disease (median: 378 µmol/L, IQR: 298–439) compared to those in remission (median: 272 µmol/L, IQR: 234–312; *p* = 0.023). CRP and calprotectin were not significant predictors of clinical remission (Figure 6A, Supplementary Material, Table S3).

##### Biochemical Remission Based on CRP

At baseline, upon adjustment for multiple comparisons, AOPP (AUC 0.787) and TAC (AUC 0.722) remained significant predictors of CRP-based remission status at Visit 3. Patients who did not respond to therapy and were CRP-active at the outcome assessment had higher baseline AOPP (median 177 µmol/L, IQR 150–191) compared to those in remission (median 131 µmol/L, IQR 101–153; *p* = 0.011), and higher baseline TAC (1.77 mM, IQR 1.59–2.30) compared to remission patients (1.49 mM, IQR 0.96–1.91; *p* = 0.041). In comparison, baseline calprotectin showed the strongest predictive performance (AUC 0.922), while CRP demonstrated acceptable predictive performance (AUC 0.744) (Figure 6B, Table 3C). However, in the UC subgroup, after adjustment for multiple comparisons, only baseline AOPP showed an outstanding discriminatory performance among oxidative stress–related biomarkers (AUC 0.916, 95% CI 0.817–1.000). Levels were significantly higher in patients with active UC (median: 169 µmol/L, IQR: 147–194; *n* = 10) compared to those in remission (median: 105 µmol/L, IQR: 76–125; *n* = 19; *p* < 0.001). No biomarkers appeared significant in the CD subgroup.

### 3.6. Principal Component Analysis (PCA) and Logistic Regression

PCA was performed to reduce dimensionality, explore redox-related patterns, and identify combinations of oxidative stress–related biomarkers that could distinguish between active disease and remission, complementing the individual ROC-based biomarker analyses. PCA was limited to Visit 3, where both biomarker levels and treatment outcomes were assessed simultaneously. Of the 61 patients who completed Visit 3, 45 with complete data across all biomarkers were included in the subsequent analysis. Due to high collinearity between total and direct bilirubin levels (Figure 3), only total bilirubin was retained in the analysis.

The first five components had eigenvalues greater than 1 (Supplementary Material, Figure S2), cumulatively explaining approximately 70% of the total variance (PC1: 21.7%, PC2: 19.2%, PC3: 11.1%, PC4: 9.5%, PC5: 8.1%). Table 4 lists the factor loadings for the first five components. PC1 showed the strongest loadings from R-SH, PC2 was characterized by TIBC and UIBC, while PC3 showed loadings from TBIL and GGT. PC4 was primarily influenced by TAC and SUA, and PC5 by MDA and SUA.

Each PC was then tested for association with remission outcomes using univariable logistic regression (Table 5). *p*-values were adjusted for multiple comparisons within each outcome (Benjamini–Hochberg). Clinical remission was associated with PC3 (OR = 2.23, *p* = 0.025) and PC5 (OR = 2.75, *p* = 0.015), while CRP remission was associated with PC1 (OR = 1.77, *p* = 0.035) and PC5 (OR = 2.51, *p* = 0.035). Full model results are shown in Table 5.

### 3.7. Improving Diagnostic Performance Through Biomarker Combinations

For each remission outcome, the biomarker with the strongest individual diagnostic performance (Table 3A, Supplementary Material, Table S1) was combined with those associated with significant PCs identified Via PCA (Table 4) and selected in logistic regression (Table 5). Only biomarkers with absolute loadings greater than 0.4 were selected from each relevant component (Table 4).

**Figure 7:**
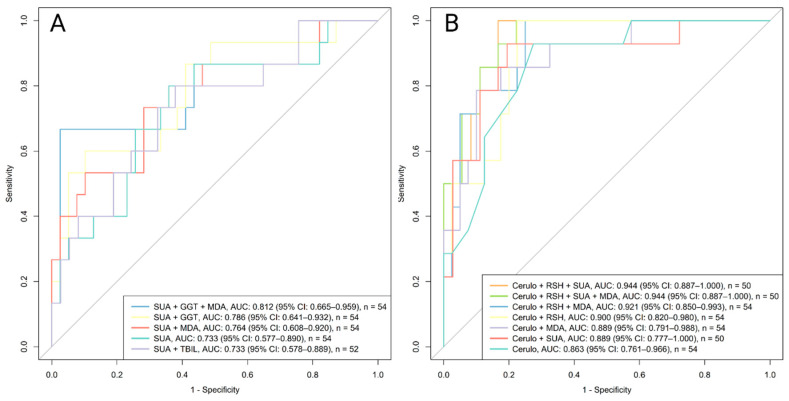
ROC curves for combined Visit 3 biomarker models predicting remission outcomes. The number of available samples varies by model and is indicated accordingly. (**A**) Clinical remission: SUA was combined with biomarkers with the highest loadings from PC3 (GGT, TBIL) and PC5 (MDA). (**B**) CRP remission: Ceruloplasmin was tested in combination with biomarkers from PC1 (R-SH) and PC5 (MDA, SUA). Abbreviations: AUC, area under the receiver operating curve; Cerulo, ceruloplasmin; CI, confidence interval; CRP, C-reactive protein; GGT, gamma-glutamyl transferase; MDA, malondialdehyde; R-SH; plasma free thiols; SUA, serum uric acid; TBIL, total bilirubin Youden, Youden Index.

Clinical remission. SUA was combined with biomarkers showing the highest loadings from PC3 (GGT, TBIL) and PC5 (MDA) (Table 4 and Table 5). The best overall performance was achieved with SUA, GGT, and MDA (AUC = 0.812, 95% CI 0.665–0.959) (Figure 7A). However, neither combination significantly improved performance compared with SUA alone (*p* = 0.274).

Biochemical (CRP) remission. Ceruloplasmin was tested in combination with biomarkers showing the highest loadings from PC1 (R-SH) and PC5 (MDA, SUA) (Table 4 and Table 5). The best combination (ceruloplasmin, R-SH, SUA) achieved an AUC of 0.944 (95% CI 0.887–1.000) (Figure 7B), but the difference versus ceruloplasmin alone was not significant (*p* = 0.181).

## 4. Discussion

**Figure 8:**
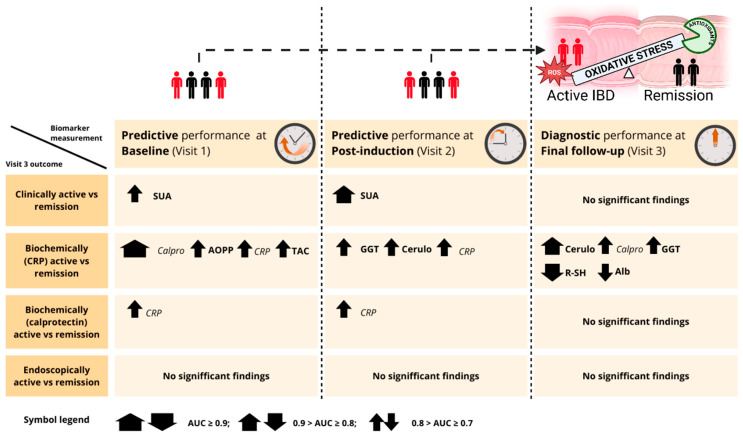
Overview of oxidative stress–related biomarkers demonstrating significant diagnostic and predictive ability to distinguish between active IBD and remission. Biomarker performance is shown at three timepoints: baseline (Visit 1), post-induction (Visit 2), and final follow-up (Visit 3). Rows correspond to disease activity definitions at Visit 3, including clinical, biochemical (CRP- and calprotectin-based), and endoscopic remission. Arrows indicate the direction of biomarker dysregulation in patients with active disease compared to those in remission. Symbol size corresponds to diagnostic or predictive strength based on AUC thresholds (see legend). Abbreviations: Alb, albumin; AOPP, advanced oxidation protein products; AUC, area under the receiver operating curve; Calpro, fecal calprotectin; Cerulo, ceruloplasmin; CRP, C-reactive protein; GGT, gamma-glutamyl transferase; R-SH, plasma free thiols; SUA, serum uric acid; TAC, total antioxidant capacity.

The role of oxidative stress in the pathogenesis of IBD has been previously investigated, with reported associations between oxidative stress-related biomarkers and disease activity [7]. However, to the best of our knowledge, this is the first study to comprehensively assess the diagnostic and predictive potential of a broad panel of oxidative stress-related biomarkers in relation to both short- and long-term treatment outcomes in IBD, including clinical, biochemical, and endoscopic remission. Summary of dysregulated oxidative stress-related biomarkers demonstrating significant diagnostic and predictive ability to distinguish between active IBD and remission is presented in Figure 8.

A comprehensive panel of 15 biomarkers was evaluated in this study, capturing antioxidants, pro-oxidants, and other indicators of oxidative damage. At baseline, when patients exhibited active disease (clinically, biochemically, or endoscopically), a clustering pattern was observed that aligned closely with the biological functions of these biomarkers (Figure 2). Acute-phase reactants reflect the systemic response to inflammation, with concentrations of positive acute-phase reactants (ferritin, ceruloplasmin) increasing and negative acute-phase reactants (albumin, transferrin, iron) decreasing [27,28]. Although transferrin was not directly measured in this study, TIBC served as a surrogate marker. In the context of IBD, active inflammation and oxidative stress lead to a decrease in antioxidants such as total bilirubin, albumin, transferrin (Via TIBC), and R-SH, while markers of oxidative damage to proteins (AOPP) and lipids (MDA) accumulate [7]. SUA is notable for its bimodal role: while it acts as an antioxidant at physiological levels, it can exert pro-inflammatory and pro-oxidative effects under pathological conditions, and is often elevated during oxidative stress [29,30,31,32]. As it is often considered a major contributor to TAC [29,31], the latter may explain the clustering of SUA and TAC we have observed in our analysis. Another member of this cluster, GGT, has also been shown to promote oxidative stress and inflammation [33,34]. These clustering patterns support the presence of oxidative stress in IBD (Figure 2).

The correlation analysis at Visit 3 revealed significant associations between oxidative stress-related biomarkers and clinical, biochemical, and endoscopic measures of disease activity (Figure 3). For example, ceruloplasmin was positively correlated with both CRP and calprotectin, while GGT also showed a significant positive correlation with CRP. In contrast, R-SH and albumin were negatively correlated with both CRP and calprotectin. R-SH was also negatively associated with rectal bleeding scores, suggesting that thiol levels may not only reflect biochemical but also clinical disease activity. Similarly, MDA and SUA were positively correlated with the stool frequency score and the number of daily liquid stools, respectively, further linking oxidative stress to the symptomatic disease activity. Meanwhile, serum iron levels showed negative correlations with CRP, stool frequency score, and the SES-CD, indicating potential relevance for assessing clinical, biochemical, and endoscopic disease activity. Collectively, our findings highlight the potential of oxidative stress-related biomarkers to study and capture diverse aspects of IBD activity.

### 4.1. Diagnostic Performance of Oxidative Stress-Related Biomarkers

#### 4.1.1. Clinical, Biochemical (Calprotectin) and Endoscopic Remission

After adjustment for multiple comparisons, none of the oxidative stress–related biomarkers were robust enough to distinguish clinically active patients from those in remission. Clinical remission itself does not necessarily imply absence of inflammation, as disease activity can persist at biochemical, endoscopic, or histologic levels [9,35]. Consistent with this observation, Rosenberg et al. reported that nearly half of patients with UC in clinical remission still exhibited endoscopic inflammation and elevated CRP levels [36]. Similarly, Bourgonje et al. showed evidence of subclinical disease activity and systemic oxidative stress in patients with CD in clinical remission [37]. This overlap likely limits the ability of systemic oxidative stress–related biomarkers to robustly separate clinically active patients from those in remission.

Oxidative stress–related biomarkers were also not reliable indicators of calprotectin-based or endoscopic remission. The limited utility of these biomarkers in IBD may reflect a broader limitation of fecal calprotectin itself, which is more reliable for detecting mucosal inflammation in colonic IBD, but appears less sensitive for identifying inflammation in small intestinal CD [38]. As our cohort was not stratified by disease location, this may have contributed to weaker biomarker performance, at least in the calprotectin-based analyses. Fecal calprotectin and endoscopy reflect localized mucosal inflammation, whereas the oxidative stress–related biomarkers in this study were measured in systemic circulation and reflect whole-body redox status rather than localized inflammation. High within-day variability in fecal calprotectin has been reported in patients with UC, with coefficients of variation around 40%. This indicates that the first bowel movement of the day may not capture the highest or lowest calprotectin levels [39]. There is also no widely accepted fecal calprotectin cutoff for remission [9]. In addition, there may be a temporal mismatch between blood sampling and mucosal activity, as systemic oxidative stress markers and mucosal inflammation may peak and/or resolve at different time points [9]. Finally, the smaller number of patients with available calprotectin (*n* = 46) and endoscopic data (*n* = 32) likely reduced statistical power, limiting the ability to detect differences between remission and active disease. Together, these factors suggest that oxidative stress–related biomarkers are likely better suited for capturing systemic inflammation rather than localized inflammation. Supporting this, albumin, ceruloplasmin, GGT, and R-SH were capable of distinguishing patients with active disease from those in remission when biochemical remission was defined by CRP.

#### 4.1.2. Biochemical (CRP) Remission

Ceruloplasmin exhibited excellent discriminatory ability in identifying patients with biochemically active IBD based on CRP levels, outperforming fecal calprotectin (Figure 4). These findings are consistent with our correlation analysis, where CRP showed the strongest association with ceruloplasmin (Figure 3). Ceruloplasmin is a positive acute-phase protein produced by hepatocytes as well as activated macrophages and monocytes, with levels increasing by approximately 50 percent in response to inflammation [28,40]. This corresponds with the significantly elevated ceruloplasmin levels observed in patients with active IBD compared to those in remission (Table 3A). Subgroup analyses reinforced these findings in UC patients, where ceruloplasmin yielded an outstanding AUC of 0.912 (95% CI 0.774–1.000). Biologically, ceruloplasmin acts as a potent antioxidant by scavenging free radicals [41] and through its ferroxidase activity, which prevents ferrous iron from participating in the Fenton reaction and generating reactive oxygen species [28,40].

R-SH also demonstrated better discriminatory ability than calprotectin, with significantly lower levels observed in active IBD patients (Table 3A, Figure 4). This aligns with the significant negative correlation observed between R-SH and CRP (Figure 3). The pool of R-SH represents an important component of plasma antioxidants, consisting of reduced forms of albumin and low-molecular-weight thiols, e.g., cysteine and glutathione. R-SH levels are reduced under oxidative stress due to the formation of thiol oxidation products [42]. Additionally, lower albumin concentrations further contribute to the observed decrease in R-SH levels. Albumin is a negative acute-phase reactant, and its levels tend to decrease in the presence of inflammation [27,28]. Moreover, factors such as malnutrition, malabsorption, and gastrointestinal losses may also lead to reduced albumin concentrations [43,44]. In line with this, at least in our study, albumin demonstrated acceptable discriminatory ability in identifying CRP-active IBD patients, with significantly lower levels observed in the active group (Table 3, Figure 4).

Similarly, GGT showed acceptable AUC values in assessing biochemically active disease (Table 3, Figure 4). The latter plays a key role in the glutathione metabolism Via production of cysteinyl glycine, which has strong reducing capacity and converts ferric iron (Fe^3+^) to ferrous iron (Fe^2+^), enabling its participation in the Fenton reaction. This process promotes the generation of reactive oxygen species and induces the oxidation of lipids and protein thiols, thereby reflecting antioxidant inadequacy. Elevated GGT levels have also been associated with cardiovascular disease and cancer [34,45]. Accordingly, we observed a significant positive correlation between GGT levels and CRP, along with significantly elevated GGT levels in patients with active IBD (Figure 3, Table 3A).

### 4.2. Oxidative Stress-Related Biomarkers as Predictors of Treatment Outcome

Beyond their diagnostic value, oxidative stress–related biomarkers may also serve as predictors of treatment outcome. To date, however, most studies in IBD have explored them primarily as indicators of disease activity rather than predictors of disease course [7,46]. In our study, patients who did not achieve clinical and CRP-based remission showed greater dysregulation of oxidative stress–related biomarkers at both baseline and the post-induction visit. Baseline dysregulation likely reflects a higher disease burden, while persistence at the post-induction visit suggests insufficient treatment response. Early assessment of oxidative stress–related biomarkers may therefore help identify patients less likely to achieve remission. Among them, serum uric acid (SUA) even outperformed established measures such as CRP and fecal calprotectin in predicting clinical remission.

Uric acid is a major plasma antioxidant that scavenges free radicals and limits their formation by chelating iron under physiological conditions. At elevated concentrations, however, it exerts pro-oxidant and pro-inflammatory effects [47,48]. Its metabolism is closely linked to the gut microbiota, and dysbiosis, as observed in IBD, may lead to elevated SUA levels [47,49]. We observed increased SUA levels in patients who failed to achieve clinical remission already at baseline, as well as at the post-induction and final follow-up visits, indicating a consistent pattern across studied time points. However, after adjusting for multiple comparisons, SUA did not reach statistical significance at the final visit. This suggests that SUA may be a more robust predictor of clinical IBD activity over time rather than a marker of immediate symptom burden. In line with this, hyperuricemia was a predictive factor for both the development and progression of type 2 diabetes, hypertension, chronic kidney disease by promoting inflammation, oxidative stress and endothelial dysfunction [47]. For CRP-based remission, ceruloplasmin and GGT demonstrated the strongest and most consistent predictive performances. They accurately predicted CRP-based remission as early as at Visit 2, whereas at baseline, after adjustment, they were borderline insignificant, which may be due to limited statistical power rather than a true absence of their association. AOPP and TAC were both elevated at baseline in patients who were CRP-active at the final assessment. Note that AOPP is a known biomarker of oxidative protein damage, generated mainly through the activity of neutrophils and macrophages, where chlorinated oxidants react with plasma proteins, primarily albumin [50]. Similarly, AOPP has been described as a predictive biomarker of progressive renal disease, not only reflecting oxidative stress but also acting as a potent mediator of monocyte activation [51]. Interestingly, increased baseline TAC levels predicted active disease, possibly reflecting an early compensatory antioxidant response to the increased oxidative stress [52]. Major contributors to plasma antioxidant capacity, such as ceruloplasmin and serum uric acid [52], also clustered with TAC in our hierarchical analysis and were elevated in patients who did not achieve remission. R-SH did not show early predictive ability, which may further support the idea that antioxidant defenses are initially upregulated before becoming depleted later in the disease course.

### 4.3. PCA and Logistic Regression

While correlation analysis allowed us to examine pairwise relationships between individual oxidative stress-related biomarkers and measures of disease activity, it does not capture broader patterns or interactions among multiple biomarkers simultaneously. To address this, PCA was conducted. Several principal components, particularly PC1, PC3, and PC5, were significantly associated with remission outcomes (Table 5). Higher PC1 scores, primarily influenced by R-SH and albumin, were associated with CRP remission. Higher PC3 scores, characterized by high bilirubin and low levels of GGT and ceruloplasmin, and higher PC5 scores, defined by low levels of MDA and SUA, were associated with clinical remission (Table 4 and Table 5). These findings suggest that patients with favorable oxidative stress-related biomarker profiles, including high levels of R-SH and albumin, and low levels of MDA, and SUA, are more likely to achieve biochemical remission based on CRP. The observed pattern aligns with earlier clustering and correlation analyses (Figure 2 and Figure 3), and is further supported by previously published studies [7]. Although combining multiple oxidative stress–related biomarkers with principal component–derived features improved AUC values for both clinical and CRP-based remission, none of the combinations significantly outperformed the strongest single biomarker data. Additionally, several of the best-performing biomarkers (e.g., GGT, SUA, and ceruloplasmin) are already measured in routine clinical practice, though not currently used to assess disease activity in IBD. Their repurposing, alongside the implementation of additional oxidative stress-related markers, could therefore provide improved tools for monitoring IBD activity.

### 4.4. Limitations

Firstly, the sample size was relatively small for some remission types, particularly endoscopic outcomes, which may have limited statistical power. Secondly, sex-based analyses were not performed due to the limited number of participants, which may affect the generalizability of the findings across sexes. Additionally, potential confounding factors such as dietary habits, smoking status, age, and other lifestyle or environmental influences on oxidative stress were not systematically captured or controlled for. Treatment regimens varied among patients, and the effect of specific drugs on redox homeostasis may have influenced biomarker levels independently of disease activity.

## 5. Conclusions

Our study is the first to provide comprehensive evidence supporting the role of oxidative stress in IBD, highlighting the potential of oxidative stress-related biomarkers for monitoring disease activity and predicting treatment outcomes. Biomarkers such as GGT, ceruloplasmin, plasma free thiols, and albumin were significantly associated with disease activity, effectively distinguishing between active IBD and remission based on CRP criteria. In addition, SUA, AOPP, GGT, ceruloplasmin, and TAC predicted clinical and CRP-based treatment outcomes at either baseline or the post-induction visit, in some cases outperforming conventional inflammatory markers such as CRP and calprotectin. However, systemic oxidative stress-related biomarkers were less effective in distinguishing remission using calprotectin cutoffs or endoscopic criteria, likely due to sample size limitations and their inability to fully capture localized inflammation.

Lastly, many of the evaluated biomarkers are already measured in routine clinical practice but are not currently used to monitor IBD activity. Repurposing these and incorporating novel oxidative stress-related biomarkers may offer a more practical, robust, and cost-effective tool for assessing disease activity and predicting treatment outcomes in IBD. Further research in larger, well-controlled multicentre studies is needed to validate these findings and establish their clinical benefits.

## Figures and Tables

**Table 1:** Levels of oxidative stress-related biomarkers, CRP, and fecal calprotectin in patients with IBD across the three visits. Median (IQR) values are shown for each biomarker at Visit 1 (*n* = 76), Visit 2 (*n* = 75), and Visit 3 (*n* = 61), along with the number of patients with available measurements.

Biomarker Median (IQR)	Visit 1: Baseline	Visit 2: Post-Induction (Week 6–12)	Visit 3: Final Follow-Up (Week 24–36)
Alb [g/L]	43 (41–45), *n* = 73	45 (43–46), *n* = 71	45 (43–46), *n* = 59
AOPP [µmol/L]	146 (115–179), *n* = 76	135 (111–176), *n* = 75	143 (115–181), *n* = 60
BILDIR [µmol/L]	3 (2–5), *n* = 67	3 (2–4), *n* = 73	4 (3–6), *n* = 58
Cerulo [g/L]	0.28 (0.25–0.32), *n* = 70	0.27 (0.25–0.31), *n* = 58	0.26 (0.22–0.29), *n* = 54
Ferritin [µg/L]	66 (27–157), *n* = 72	59 (28–133), *n* = 70	49 (29–106), *n* = 59
GGT [µkat/L]	0.36 (0.25–0.58), *n* = 74	0.35 (0.26–0.56), *n* = 73	0.31 (0.23–0.51), *n* = 61
Hb [g/L]	135 (125–146), *n* = 73	136 (125–149), *n* = 72	140 (128–152), *n* = 61
Iron [µmol/L]	14.5 (10.0–19.0), *n* = 73	15.7 (10.4–20.0), *n* = 74	16.7 (12.8–23.3), *n* = 61
MDA [µmol/L]	3.31 (2.90–3.77), *n* = 76	3.16 (2.80–3.93), *n* = 75	3.29 (2.96–3.76), *n* = 60
R-SH [µmol/L]	445 (386–511), *n* = 76	438 (381–485), *n* = 75	481 (420–545), *n* = 60
SUA [µmol/L]	293 (237–349), *n* = 70	294 (247–366), *n* = 64	311 (261–361), *n* = 54
TAC [mM]	1.62 (1.05–2.09), *n* = 76	1.40 (0.97–1.79), *n* = 75	1.49 (0.75–2.04), *n* = 61
TBIL [µmol/L]	10 (7–14), *n* = 67	10 (7–14), *n* = 73	9 (7–15), *n* = 58
TIBC [µmol/L]	50.3 (44.4–57.1), *n* = 72	51.1 (47.6–57.6), *n* = 69	52.9 (47.0–57.5), *n* = 58
UIBC [µmol/L]	34.7 (29.8–42.4), *n* = 72	35.9 (30.8–43.8), *n* = 71	33.1 (27.6–41.1), *n* = 59
CALPRO [mg/kg]	309 (77–702), *n* = 24	129 (38–371), *n* = 24	62 (28–143), *n* = 46
CRP [mg/L]	0 (0–6), *n* = 74	0 (0–4), *n* = 74	0 (0–0), *n* = 61

Abbreviations: Alb, albumin; AOPP, advanced oxidation protein products; BILDIR, direct bilirubin; CALPRO, fecal calprotectin; Cerulo, ceruloplasmin; CRP, C-reactive protein; GGT, gamma-glutamyl transferase; Hb, hemoglobin; IQR, interquartile range; MDA, malondialdehyde; n, number of samples available; R-SH, plasma free thiols; SUA, serum uric acid; TAC, total antioxidant capacity; TBIL, total bilirubin; TIBC, total iron-binding capacity; UIBC, unsaturated iron-binding capacity.

**Table 2:** Clinical, biochemical (CRP and calprotectin), and endoscopic remission rates across study visits. Values are reported as the number of patients in remission over the number with complete outcome data (N_R_/N_T_), with corresponding percentages shown in parentheses. The denominator varies between visits due to treatment discontinuation and within visits due to missing outcome data.

	Visit 1: Baseline	Visit 2: Post-Induction (Week 6–12)	Visit 3: Final Follow-Up (Week 24–36)
Clinical remission, N_R_/N_T_ (%)	25/76 (32.9%)	42/74 (56.8%)	44/61 (72.1%)
CRP remission, N_R_/N_T_ (%)	44/74 (59.5%)	55/74 (74.3%)	46/61 (75.4%)
Calprotectin remission, N_R_/N_T_ (%)	7/24 (29.2%)	11/24 (45.8%)	30/46 (65.2%)
Endoscopic remission, N_R_/N_T_ (%)	0/39 (0%)	3/11 (27.3%)	15/32 (46.9%)

Abbreviations: CRP, C-reactive protein; N_R_, number of patients in remission; N_T_, total number of patients with complete outcome data.

**Table 3:** Diagnostic and predictive performance of oxidative stress–related biomarkers at the final visit (A), visit 2 (B), and baseline (C) for CRP remission in patients with IBD. Biomarkers were evaluated using ROC curve analysis with AUC values and 95% confidence intervals. *p*-values are from Mann–Whitney U tests comparing biomarker levels between active disease and remission. *p*-values were adjusted for multiple comparisons within each outcome (Benjamini–Hochberg). Statistically significant results (*p* < 0.05) are shown in bold. Cut-off signs (>, <) indicate whether higher or lower biomarker levels were associated with remission.

Biomarker	(A) Assessment of CRP Remission Based on Final Follow-Up Biomarker Measurements						(B) Prediction of CRP Remission Based on Visit 2 Biomarker Measurements						(C) Prediction of CRP Remission Based on Baseline Biomarker Measurements					
	N_A_/N_R_	AUC [95% CI]	Cutoff	SN [%]	SP [%]	*p*	N_A_/N_R_	AUC [95% CI]	Cutoff	SN [%]	SP [%]	*p*	N_A_/N_R_	AUC [95% CI]	Cutoff	SN [%]	SP [%]	*p*
Alb [g/L]	**14/45**	**0.714 [0.576–0.852]**	**>45.5**	**92.9**	**51.1**	**0.048**	14/43	0.708 [0.545–0.870]	>44.5	78.6	58.1	0.086	14/44	0.464 [0.291–0.637]	<42.5	71.4	34.1	0.868
AOPP [µmol/L]	15/45	0.552 [0.378–0.725]	<166	53.3	68.9	0.809	15/46	0.642 [0.462–0.822]	<176	53.3	82.6	0.290	**15/46**	**0.787 [0.671–0.903]**	**<136**	**93.3**	**56.5**	**0.011**
BILDIR [µmol/L]	15/43	0.536 [0.383–0.688]	<3.5	66.7	48.8	0.886	15/44	0.499 [0.341–0.658]	<2.5	73.3	31.8	1.000	12/40	0.583 [0.429–0.738]	<2.5	91.7	35	0.640
Cerulo [g/L]	**14/40**	**0.863 [0.761–0.966]**	**<0.27**	**92.9**	**72.5**	**<0.001**	**14/32**	**0.746 [0.571–0.920]**	**<0.3**	**57.1**	**84.4**	**0.049**	14/42	0.707 [0.553–0.861]	<0.30	78.6	66.7	0.061
Ferritin [µg/L]	14/45	0.483 [0.273–0.692]	>15.5	21.4	95.6	0.887	14/43	0.478 [0.274–0.682]	>59.5	57.1	55.8	0.953	13/44	0.608 [0.411–0.806]	<166	46.2	86.4	0.514
GGT [µkat/L]	**15/46**	**0.725 [0.590–0.861]**	**<0.27**	**93.3**	**45.7**	**0.038**	**15/44**	**0.764 [0.632–0.895]**	**<0.395**	**73.3**	**75**	**0.021**	15/44	0.711 [0.550–0.873]	<0.38	73.3	70.5	0.053
Hb [g/L]	15/46	0.668 [0.505–0.831]	>131	60	78.3	0.106	15/43	0.589 [0.395–0.784]	>128	53.3	72.1	0.588	15/44	0.533 [0.347–0.719]	>126.5	46.7	77.3	0.868
Iron [µmol/L]	15/46	0.670 [0.501–0.839]	>10.8	40	89.1	0.106	15/45	0.625 [0.444–0.806]	>14.75	66.7	64.4	0.322	14/44	0.541 [0.378–0.703]	<13.15	85.7	40.9	0.868
MDA [µmol/L]	15/45	0.691 [0.535–0.848]	<3.50	66.7	71.1	0.075	15/46	0.571 [0.395–0.747]	<3.00	80	45.7	0.708	15/46	0.495 [0.304–0.686]	<3.89	33.3	80.4	0.977
R-SH [µmol/L]	**15/45**	**0.800 [0.676–0.924]**	**>468**	**80**	**68.9**	**0.003**	15/46	0.545 [0.367–0.723]	<456	66.7	60.9	0.868	15/46	0.616 [0.445–0.787]	>473	80	43.5	0.449
SUA [µmol/L]	15/39	0.586 [0.402–0.771]	<310	66.7	53.8	0.538	14/37	0.519 [0.323–0.716]	<435	28.6	91.9	0.953	15/41	0.554 [0.368–0.741]	<278.5	73.3	51.2	0.837
TAC [mM]	15/46	0.487 [0.316–0.658]	<1.43	60	50	0.887	15/46	0.648 [0.490–0.806]	<1.59	60	67.4	0.290	**15/46**	**0.722 [0.576–0.868]**	**<2.25**	**40**	**95.7**	**0.041**
TBIL [µmol/L]	15/43	0.526 [0.371–0.680]	<6.5	93.3	20.9	0.886	15/44	0.495 [0.334–0.657]	<7.5	73.3	31.8	1.000	12/40	0.605 [0.450–0.761]	<8.5	83.3	45	0.521
TIBC [µmol/L]	13/45	0.529 [0.339–0.719]	>53.9	69.2	48.9	0.886	13/42	0.549 [0.357–0.740]	>50.5	69.2	50	0.868	13/44	0.503 [0.311–0.696]	<57	30.8	81.8	0.977
UIBC [µmol/L]	14/45	0.587 [0.415–0.758]	<31.5	71.4	48.9	0.538	14/43	0.521 [0.321–0.720]	<39.5	42.9	76.7	0.953	13/44	0.485 [0.291–0.679]	<34.8	53.8	56.8	0.977
CALPRO [mg/kg]	**15/31**	**0.743 [0.587–0.899]**	**<125**	**60**	**87.1**	**0.038**	6/15	0.722 [0.435–1.000]	<292	66.7	86.7	0.307	**4/16**	**0.922 [0.796–1]**	**<528**	**100**	**81.2**	**0.041**
CRP [mg/L]	**15/46**	**1.000 [1.000–1.000]**	**<5**	**100**	**100**	**<0.001**	**15/45**	**0.713 [0.572–0.854]**	**<2.5**	**60**	**84.4**	**0.021**	**15/44**	**0.744 [0.602–0.886]**	**<2.5**	**73.3**	**72.7**	**0.012**

Abbreviations: Alb, albumin; AOPP, advanced oxidation protein products; AUC, area under the receiver operating curve; BILDIR, direct bilirubin; CALPRO, calprotectin; Cerulo, ceruloplasmin; CI, 95% confidence interval for area under the curve; CRP, C-reactive protein; cutoff, threshold that best separates patients in remission vs. active disease based on Youden index (the direction of the inequality (>, <) indicates the biomarker value associated with remission); GGT, gamma-glutamyl transferase; Hb, hemoglobin; MDA, malondialdehyde; n, total number of patients with available outcome data; NA, number of patients with active disease included in the analysis; NR, number of patients in remission included in the analysis; p, result of the Mann–Whitney U test comparing biomarker levels between patients with active disease and those in remission, adjusted for multiple comparisons using the Benjamini–Hochberg procedure; R-SH, plasma free thiols; SN, sensitivity (%); SP, specificity (%); SUA, serum uric acid; TAC, total antioxidant capacity; TBIL, total bilirubin; TIBC, total iron-binding capacity; UIBC, unsaturated iron-binding capacity.

**Table 4:** Principal component loadings for 14 oxidative stress-related biomarkers across the first five components identified by PCA (*n* = 45). Loadings represent the contribution of each biomarker to the respective principal component. The percentage in parentheses indicates the proportion of total variance explained by each component.

Biomarker	PC1 (21.7%)	PC2 (19.2%)	PC3 (11.1%)	PC4 (9.5%)	PC5 (8.1%)
Alb	0.3501	0.0146	0.2558	0.1738	0.0674
AOPP	0.3053	−0.2069	−0.3274	0.0635	−0.0145
Cerulo	−0.2787	−0.092	−0.3237	0.2937	−0.2319
Ferritin	0.0585	0.37	−0.322	−0.1793	−0.1903
GGT	0.0436	0.2148	**−** **0.4372**	0.1315	−0.1933
Hb	0.3722	0.1596	−0.2402	−0.3103	0.129
Iron	0.2868	0.36	0.2154	0.2532	−0.2045
MDA	−0.266	−0.0338	0.1743	−0.0876	**−** **0.6403**
R-SH	**0.4879**	−0.0601	−0.0778	0.1895	0.021
SUA	0.2487	−0.1293	−0.1618	**−** **0.4382**	**−** **0.4844**
TAC	0.1319	−0.2029	−0.0291	**0.5993**	−0.2879
TBIL	0.1748	0.109	**0.4936**	−0.187	−0.252
TIBC	0.2477	**−** **0.4607**	0.1056	−0.034	−0.1226
UIBC	−0.023	**−** **0.5689**	−0.0736	−0.1971	0.0542

Important loadings (absolute value of coefficient above 0.4) are in bold. Alb, albumin; AOPP, advanced oxidation protein products; Cerulo, ceruloplasmin; GGT, gamma-glutamyl transferase; Hb, hemoglobin; MDA, malondialdehyde; PC, principal component; R-SH, plasma free thiols; SUA, serum uric acid; TAC, total antioxidant capacity; TBIL, total bilirubin; TIBC, total iron-binding capacity; UIBC, unsaturated iron-binding capacity.

**Table 5:** Univariable Logistic Regression Analysis of Predictors of Treatment Outcome (*n* = 45).

PC	Clinical Remission (*n* = 45)				CRP Remission (*n* = 45)				Calprotectin Remission (*n* = 36)				Endoscopic Remission (*n* = 26)			
	N_A_/N_R_	OR	95% CI	*p*	N_A_/N_R_	OR	95% CI	*p*	N_A_/N_R_	OR	95% CI	*p*	N_A_/N_R_	OR	95% CI	*p*
PC1	14/ 31	0.92	0.63, 1.34	0.658	13/ 32	1.77	1.11, 3.27	0.035	11/ 25	1.28	0.83, 2.14	0.458	14/ 12	0.69	0.29, 1.51	0.888
PC2	14/ 31	1.14	0.77, 1.70	0.631	13/ 32	1.02	0.68, 1.52	0.918	11/ 25	1.1	0.73, 1.66	0.788	14/ 12	0.93	0.59, 1.46	0.952
PC3	14/ 31	2.23	1.19, 4.94	0.025	13/ 32	1.87	1.03, 3.87	0.065	11/ 25	1.93	1.01, 4.50	0.115	14/ 12	1.02	0.56, 1.87	0.952
PC4	14/ 31	1.31	0.75, 2.42	0.583	13/ 32	1.19	0.67, 2.19	0.691	11/ 25	2.13	1.03, 5.32	0.115	14/ 12	1.58	0.74, 3.87	0.888
PC5	14/ 31	2.75	1.37, 6.60	0.015	13/ 32	2.51	1.26, 5.81	0.035	11/ 25	0.97	0.46, 1.99	0.936	14/ 12	1.09	0.49, 2.45	0.952

Abbreviations: CI, Confidence Interval; Comp., principal component; CRP, C-reactive protein; n, number of patients with full biomarker and outcome data; N_A_, number of patients with active disease included in the analysis; N_R_, number of patients in remission included in the analysis; OR, Odds Ratio; *p*, *p*-value from a log-likelihood ratio test assessing the association of each principal component with the remission outcome, adjusted for multiple comparisons using the Benjamini–Hochberg procedure; PC, Principal component.

## Data Availability

All necessary data for assessing the paper’s conclusions are provided in the main text and Supplementary Materials. Additional data can be obtained from the corresponding author upon request.

## Supplementary Materials

The following supporting information can be downloaded at https://www.mdpi.com/article/10.3390/antiox14101183/s1: Table S1. Diagnostic performances of oxidative stress-related biomarkers at Visit 3 in relation to various remission outcomes in patients with IBD. Table S2. Predictive performances of oxidative stress-related biomarkers at Visit 2 in relation to various remission outcomes in patients with IBD. Table S3. Predictive performances of oxidative stress-related biomarkers at baseline in relation to various remission outcomes in patients with IBD. Figure S1. Heatmap showing hierarchical clustering of 15 oxidative stress-related biomarkers in patients with IBD at baseline, annotated by drug, biologic-naïve status, smoking status, sex, and IBD subtype (CD vs. UC). Figure S2. Scree plot showing the eigenvalues of the principal components (dimensions).

## Abbreviations

The following abbreviations are used in this manuscript:

AOPP	Advanced oxidation protein products
AUC	Area under the receiver operating characteristic curve
CD	Crohn’s disease
CI	Confidence interval
CRP	C-reactive protein
GGT	Gamma-glutamyl transferase
IBD	Inflammatory bowel disease
IQR	Interquartile range
MDA	Malondialdehyde
R-SH	Plasma free thiols
SUA	Serum uric acid
TAC	Total antioxidant capacity
UC	Ulcerative colitis
