# Supplementary material for: Oxidative Stress-Related Biomarkers in Inflammatory Bowel Disease: Dual Tools for Remission Assessment and Prediction of Treatment Outcome
Source: Antioxidants (Basel). 2025 Sep 28;14(10):1183. doi: 10.3390/antiox14101183 (PMC12561064; doi:10.3390/antiox14101183)
Supplement: Supplementary file 1 [file antioxidants-14-01183-s001.zip › antioxidants-3870421-supplementary.pdf]

Table S1. Diagnostic performances of oxidative stress-related biomarkers at Visit 3 in relation to various remission outcomes in patients with IBD. Each biomarker is evaluated using the ROC curve analysis with corresponding AUC values and 95% confidence intervals. Comparisons are shown for clinical remission (n = 61), biochemical calprotectin remission (n = 46), and endoscopic remission (n = 32). P-values are from Mann–Whitney U tests comparing biomarker levels between active disease and remission and were adjusted for multiple comparisons within each outcome (Benjamini–Hochberg). Statistically significant results ($p < 0.05$) are shown in bold. The direction of the inequality sign (>, <) in the cutoff values indicates whether higher or lower biomarker levels are associated with remission.

**Biomarker at Visit 3**	**Clinical remission (n=61)**						**Biochemical (Calprotectin) remission (n = 46)**						**Endoscopic remission (n=32)**					
	**N_A/ N_R**	**AUC [95% CI]**	**Cutoff**	**SN [%]**	**SP [%]**	**p**	**N_A/ N_R**	**AUC [95% CI]**	**Cutoff**	**SN [%]**	**SP [%]**	**p**	**N_A/ N_R**	**AUC [95% CI]**	**Cutoff**	**SN [%]**	**SP [%]**	**p**
Alb [g/L]	17/ 42	0.612 [0.446–0.778]	>42.5	41.2	83.3	0.389	14/ 30	0.667 [0.492–0.842]	>44.5	71.4	60	0.306	15/ 15	0.627 [0.425–0.829]	<40.5	100	20	0.816
AOPP [µmol/L]	17/ 43	0.564 [0.383–0.745]	<164	58.8	67.4	0.638	16/ 30	0.493 [0.297–0.688]	>131	50	73.3	0.991	17/ 15	0.569 [0.349–0.789]	>159	82.4	53.3	1.000
BILDIR [µmol/L]	17/ 41	0.458 [0.301–0.615]	<8.5	11.8	92.7	0.744	15/ 30	0.592 [0.425–0.759]	>7.5	100	20	0.602	17/ 15	0.500 [0.295–0.705]	<9.5	11.8	100	1.000
Cerulo [g/L]	15/ 39	0.613 [0.456–0.770]	<0.25	80	48.7	0.389	15/ 27	0.631 [0.449–0.812]	<0.29	46.7	77.8	0.446	17/ 13	0.638 [0.420–0.856]	>0.24	52.9	84.6	0.816
Ferritin [µg/L]	17/ 42	0.468 [0.277–0.658]	>15.5	17.6	95.2	0.800	15/ 30	0.463 [0.278–0.648]	<20	86.7	20	0.933	15/ 15	0.511 [0.294–0.728]	>32.5	53.3	66.7	1.000
GGT [µkat/L]	17/ 44	0.682 [0.523–0.841]	<0.33	76.5	61.4	0.247	16/ 30	0.597 [0.421–0.773]	<0.46	56.2	70	0.602	17/ 15	0.631 [0.424–0.838]	<0.3	64.7	73.3	0.816
Hb [g/L]	17/ 44	0.497 [0.321–0.674]	>134.5	47.1	65.9	0.981	16/ 30	0.507 [0.327–0.688]	<123.5	87.5	23.3	0.991	17/ 15	0.633 [0.434–0.833]	<146.5	47.1	86.7	0.816
Iron [µmol/L]	17/ 44	0.638 [0.476–0.801]	>12.7	47.1	84.1	0.333	16/ 30	0.588 [0.411–0.764]	>19.5	68.8	50	0.602	17/ 15	0.506 [0.297–0.714]	<27.5	23.5	93.3	1.000
MDA [µmol/L]	17/ 43	0.609 [0.457–0.761]	<3.08	82.4	46.5	0.389	16/ 30	0.498 [0.318–0.678]	<3.44	56.2	63.3	0.991	17/ 15	0.529 [0.319–0.739]	<2.87	88.2	26.7	1.000
R-SH [µmol/L]	17/ 43	0.655 [0.485–0.826]	>468	70.6	67.4	0.306	16/ 30	0.673 [0.486–0.860]	>394	37.5	100	0.301	17/ 15	0.533 [0.319–0.748]	>464	64.7	60	1.000

Biomarker at Visit 3	Clinical remission (n=61)						Biochemical (Calprotectin) remission (n = 46)						Endoscopic remission (n=32)					
	N_A/ N_R	AUC [95% CI]	Cutoff	SN [%]	SP [%]	p	N_A/ N_R	AUC [95% CI]	Cutoff	SN [%]	SP [%]	p	N_A/ N_R	AUC [95% CI]	Cutoff	SN [%]	SP [%]	p
SUA [µmol/L]	15/ 39	0.733 [0.577–0.890]	<312	80	64.1	0.153	15/ 26	0.638 [0.457–0.820]	<370	40	88.5	0.446	17/ 13	0.593 [0.377–0.809]	<325	47.1	92.3	1.000
TAC [mM]	17/ 44	0.591 [0.429–0.753]	<1.58	58.8	65.9	0.428	16/ 30	0.699 [0.530–0.868]	>1.18	56.2	83.3	0.228	17/ 15	0.582 [0.376–0.789]	>1.59	70.6	53.3	1.000
TBIL [µmol/L]	17/ 41	0.485 [0.323–0.647]	<8.5	70.6	36.6	0.918	15/ 30	0.439 [0.268–0.610]	<6.5	86.7	23.3	0.821	17/ 15	0.478 [0.272–0.685]	<26	17.6	93.3	1.000
TIBC [µmol/L]	16/ 42	0.557 [0.376–0.738]	<64.3	25	97.6	0.666	14/ 30	0.524 [0.320–0.728]	>46.75	28.6	90	0.991	15/ 15	0.507 [0.282–0.731]	<53.9	60	66.7	1.000
UIBC [µmol/L]	17/ 42	0.606 [0.435–0.778]	<49.8	29.4	95.2	0.389	15/ 30	0.462 [0.276–0.648]	>27.6	33.3	76.7	0.933	15/ 15	0.516 [0.298–0.733]	>40.9	66.7	46.7	1.000
CALPRO [mg/kg]	17/ 29	0.601 [0.430–0.773]	<51	70.6	55.2	0.428	**16/ 30**	**1.000 [1.000–1.000]**	**<100**	**100**	**100**	**<0.001**	13/ 12	0.785 [0.583–0.988]	<57	84.6	75	0.280
CRP [mg/L]	17/ 44	0.614 [0.478–0.749]	<6.5	41.2	86.4	0.306	16/ 30	0.680 [0.531–0.830]	<3	56.2	80	0.228	17/ 15	0.525 [0.362–0.689]	<6.5	29.4	86.7	1.000

Abbreviations: Alb, albumin; AOPP, advanced oxidation protein products; AUC, area under the receiver operating curve; BILDIR, direct bilirubin; CALPRO, calprotectin; Cerulo, ceruloplasmin; CI, 95% confidence interval for area under the curve; CRP, C-reactive protein; cutoff, threshold that best separates patients in remission vs. active disease based on Youden index (the direction of the inequality (>, <) indicates the biomarker value associated with remission); GGT, gamma-glutamyl transferase; Hb, hemoglobin; MDA, malondialdehyde; n, total number of patients with available outcome data; N_A, number of patients with active disease included in the analysis; N_R, number of patients in remission included in the analysis; p, result of the Mann–Whitney U test comparing biomarker levels between patients with active disease and those in remission, adjusted for multiple comparisons using the Benjamini–Hochberg procedure; R-SH, plasma free thiols; SN, sensitivity (%); SP, specificity (%); SUA, serum uric acid; TAC, total antioxidant capacity; TBIL, total bilirubin; TIBC, total iron-binding capacity; UIBC, unsaturated iron-binding capacity.

Table S2. Predictive performances of oxidative stress-related biomarkers at Visit 2 in relation to various remission outcomes in patients with IBD. Each biomarker is evaluated using the ROC curve analysis with corresponding AUC values and 95% confidence intervals. Comparisons are shown for clinical remission (n = 61), biochemical calprotectin remission (n = 46), and endoscopic remission (n = 32). P-values are from Mann–Whitney U tests comparing biomarker levels between active disease and remission and were adjusted for multiple comparisons within each outcome (Benjamini–Hochberg). Statistically significant results ($p < 0.05$) are shown in bold. The direction of the inequality sign (>, <) in the cutoff values indicates whether higher or lower biomarker levels are associated with remission.

Biomarker at Visit 2	Clinical remission (n=61)						Biochemical (Calprotectin) remission (n = 46)						Endoscopic remission (n=32)					
	N_A/ N_R	AUC [95% CI]	Cutoff	SN [%]	SP [%]	p	N_A/ N_R	AUC [95% CI]	Cutoff	SN [%]	SP [%]	p	N_A/ N_R	AUC [95% CI]	Cutoff	SN [%]	SP [%]	p
Alb [g/L]	15/ 42	0.597 [0.416–0.778]	>41.5	40	85.7	0.917	15/ 28	0.539 [0.351–0.728]	>44.5	53.3	57.1	0.828	16/ 13	0.601 [0.385–0.817]	<45.5	50	76.9	0.896
AOPP [µmol/L]	17/ 44	0.428 [0.262–0.594]	>126	47.1	61.4	0.919	16/ 30	0.646 [0.472–0.820]	<125	81.2	53.3	0.370	17/ 15	0.698 [0.492–0.904]	<120	88.2	66.7	0.556
BILDIR [µmol/L]	16/ 43	0.499 [0.336–0.662]	<3.5	50	58.1	1.000	15/ 29	0.494 [0.324–0.665]	<2.5	73.3	34.5	1.000	17/ 14	0.567 [0.364–0.771]	>3.5	58.8	57.1	0.896
Cerulo [g/L]	12/ 34	0.525 [0.352–0.697]	<0.25	91.7	29.4	0.985	14/ 20	0.598 [0.380–0.817]	<0.3	57.1	80	0.615	11/ 12	0.466 [0.214–0.718]	<0.33	27.3	83.3	0.912
Ferritin [µg/L]	15/ 42	0.544 [0.352–0.737]	<180.5	33.3	88.1	0.985	15/ 28	0.537 [0.345–0.728]	>60.5	66.7	57.1	0.828	16/ 13	0.543 [0.325–0.762]	<55.5	56.2	61.5	0.912
GGT [µkat/L]	16/ 43	0.711 [0.540–0.883]	<0.41	68.8	79.1	0.114	16/ 29	0.573 [0.397–0.749]	<0.265	81.2	37.9	0.659	17/ 13	0.629 [0.416–0.842]	<0.36	52.9	76.9	0.819
Hb [g/L]	16/ 42	0.560 [0.372–0.748]	<144	56.2	73.8	0.919	16/ 29	0.532 [0.347–0.718]	<141.5	56.2	65.5	0.828	16/ 13	0.596 [0.369–0.824]	<136.5	56.2	84.6	0.896
Iron [µmol/L]	16/ 44	0.565 [0.399–0.732]	>18.2	75	40.9	0.919	16/ 29	0.733 [0.581–0.885]	>15.2	75	69	0.092	17/ 14	0.506 [0.293–0.720]	>17.2	76.5	35.7	0.968
MDA [µmol/L]	17/ 44	0.606 [0.435–0.778]	<3.90	47.1	81.8	0.866	16/ 30	0.669 [0.496–0.841]	>3.64	87.5	46.7	0.325	17/ 15	0.645 [0.449–0.842]	>3.13	64.7	66.7	0.714
R-SH [µmol/L]	17/ 44	0.521 [0.341–0.701]	>348	23.5	93.2	0.985	16/ 30	0.554 [0.359–0.749]	>395	37.5	83.3	0.794	17/ 15	0.490 [0.280–0.700]	<597	17.6	100	0.968
SUA [µmol/L]	**13/ 38**	**0.816 [0.684–0.947]**	**<423**	**53.8**	**94.7**	**0.013**	15/ 23	0.607 [0.417–0.797]	<260.5	86.7	39.1	0.615	15/ 11	0.718 [0.504–0.932]	<259	80	72.7	0.556

TAC [mM]	17/ 44	0.618 [0.458–0.777]	<1.66	52.9	75	0.866	16/ 30	0.583 [0.405–0.762]	<1.55	56.2	66.7	0.615	17/ 15	0.669 [0.467–0.871]	<1.16	82.4	60	0.615
TBIL [μmol/L]	16/ 43	0.498 [0.331–0.665]	>9.5	56.2	55.8	1.000	15/ 29	0.589 [0.418–0.759]	>13	86.7	37.9	0.615	17/ 14	0.571 [0.358–0.784]	>9.5	58.8	78.6	0.896
TIBC [μmol/L]	14/ 41	0.523 [0.328–0.718]	<57.1	35.7	82.9	0.985	14/ 27	0.500 [0.307–0.693]	<54.2	42.9	70.4	1.000	15/ 13	0.536 [0.311–0.761]	>46.7	33.3	84.6	0.912
UIBC [μmol/L]	15/ 42	0.581 [0.403–0.759]	<39.5	46.7	78.6	0.919	15/ 28	0.667 [0.494–0.840]	<33.25	86.7	53.6	0.325	16/ 13	0.541 [0.321–0.760]	<31.45	75	38.5	0.912
CALPRO [mg/kg]	5/ 16	0.513 [0.222–0.803]	<179	60	62.5	1.000	6/ 10	0.683 [0.401–0.965]	<292	66.7	80	0.615	8/ 6	0.625 [0.227–1.000]	<137.5	87.5	66.7	0.896
CRP [mg/L]	16/44	0.472 [0.350–0.593]	<0	100	0	0.985	**16/ 29**	**0.714 [0.574–0.855]**	**<2.5**	**56.2**	**89.7**	**0.044**	17/ 14	0.534 [0.360–0.707]	<21.5	11.8	100	0.912

Abbreviations: Alb, albumin; AOPP, advanced oxidation protein products; AUC, area under the receiver operating curve; BILDIR, direct bilirubin; CALPRO, calprotectin; Cerulo, ceruloplasmin; CI, 95% confidence interval for area under the curve; CRP, C-reactive protein; cutoff, threshold that best separates patients in remission vs. active disease based on Youden index (the direction of the inequality (>, <) indicates the biomarker value associated with remission); GGT, gamma-glutamyl transferase; Hb, haemoglobin; IBDQ, inflammatory bowel disease questionnaire; MDA, malondialdehyde; n, total number of patients with available outcome data; N_A, number of patients with active disease included in the analysis; N_R, number of patients in remission included in the analysis; p, result of the Mann–Whitney U test comparing biomarker levels between patients with active disease and those in remission, adjusted for multiple comparisons using the Benjamini–Hochberg procedure; R-SH, plasma free thiols; SN, sensitivity; SP, specificity; SUA, serum uric acid; TAC, total antioxidant capacity; TBIL, total bilirubin; TIBC, total iron-binding capacity; UIBC, unsaturated iron-binding capacity.

Table S3. Predictive performances of oxidative stress-related biomarkers at baseline in relation to various remission outcomes in patients with IBD. Each biomarker is evaluated using the ROC curve analysis with corresponding AUC values and 95% confidence intervals. Comparisons are shown for clinical remission (n = 61), biochemical calprotectin remission (n = 46), and endoscopic remission (n = 32). P-values are from Mann–Whitney U tests comparing biomarker levels between active disease and remission and were adjusted for multiple comparisons within each outcome (Benjamini–Hochberg). Statistically significant results (p < 0.05) are shown in bold. The direction of the inequality sign (>, <) in the cutoff values indicates whether higher or lower biomarker levels are associated with remission.

**Biomarker at baseline**	**Clinical remission (n=61)**						**Biochemical (Calprotectin) remission (n = 46)**						**Endoscopic remission (n=32)**					
	**N_A/ N_R**	**AUC [95% CI]**	**Cutoff**	**SN [%]**	**SP [%]**	**p**	**N_A/ N_R**	**AUC [95% CI]**	**Cutoff**	**SN [%]**	**SP [%]**	**p**	**N_A/ N_R**	**AUC [95% CI]**	**Cutoff**	**SN [%]**	**SP [%]**	**p**
**Alb [g/L]**	17/ 41	0.411 [0.258–0.564]	<42.5	70.6	34.1	0.547	15/ 29	0.554 [0.372–0.736]	>43.5	66.7	55.2	0.685	16/ 15	0.598 [0.388–0.808]	<42.5	81.2	46.7	0.763
**AOPP [µmol/L]**	17/ 44	0.723 [0.591–0.856]	<136	88.2	56.8	0.056	16/ 30	0.738 [0.586–0.889]	<148	87.5	70	0.067	17/ 15	0.682 [0.483–0.882]	<147	58.8	80	0.700
**BILDIR [µmol/L]**	16/ 36	0.628 [0.480–0.775]	<2.5	93.8	38.9	0.448	14/ 25	0.400 [0.227–0.573]	<0	100	0	0.608	16/ 13	0.418 [0.214–0.623]	<6.5	12.5	92.3	0.763
**Cerulo [g/L]**	17/ 39	0.620 [0.476–0.764]	<0.25	100	35.9	0.448	15/ 28	0.580 [0.390–0.770]	<0.30	60	64.3	0.608	15/ 15	0.547 [0.331–0.762]	>0.34	93.3	26.7	0.921
**Ferritin [µg/L]**	16/ 41	0.583 [0.400–0.766]	<158.5	43.8	82.9	0.574	14/ 29	0.599 [0.416–0.781]	<41.5	78.6	44.8	0.608	16/ 15	0.488 [0.275–0.700]	<41.5	62.5	53.3	0.921
**GGT [µkat/L]**	17/ 42	0.708 [0.551–0.865]	<0.37	70.6	64.3	0.075	16/ 29	0.623 [0.449–0.797]	<0.30	87.5	37.9	0.608	17/ 15	0.606 [0.395–0.816]	<0.29	76.5	60	0.763
**Hb [g/L]**	17/ 42	0.548 [0.377–0.720]	<136	64.7	57.1	0.685	16/ 29	0.520 [0.328–0.712]	<142	50	69	0.831	17/ 15	0.625 [0.426–0.825]	<139	52.9	80	0.763
**Iron [µmol/L]**	17/ 41	0.487 [0.325–0.650]	>23.3	94.1	14.6	0.885	15/ 29	0.576 [0.392–0.760]	>16.45	73.3	51.7	0.608	16/ 15	0.538 [0.326–0.749]	<20.1	31.2	86.7	0.921
**MDA [µmol/L]**	17/ 44	0.565 [0.397–0.732]	<3.55	47.1	72.7	0.646	16/ 30	0.586 [0.411–0.762]	>2.97	43.8	80	0.608	17/ 15	0.692 [0.497–0.887]	>3.48	88.2	53.3	0.700
**R-SH [µmol/L]**	17/ 44	0.544 [0.387–0.702]	>443	58.8	61.4	0.685	16/ 30	0.521 [0.329–0.712]	>446	62.5	60	0.831	17/ 15	0.588 [0.368–0.809]	<367	94.1	40	0.763

**SUA [μmol/L]**	**16/40**	**0.777 [0.627–0.927]**	**<340.5**	**62.5**	**87.5**	**0.023**	15/27	0.617 [0.423–0.812]	<278.5	80	55.6	0.608	17/14	0.607 [0.395–0.819]	<305.5	41.2	92.9	0.763
**TAC [mM]**	17/44	0.672 [0.526–0.819]	<1.46	88.2	52.3	0.161	16/30	0.554 [0.369–0.740]	<1.76	56.2	63.3	0.685	17/15	0.549 [0.342–0.756]	>0.997	29.4	86.7	0.921
**TBIL [μmol/L]**	16/36	0.610 [0.455–0.766]	<7.5	93.8	38.9	0.511	14/25	0.420 [0.239–0.601]	<6.5	92.9	16	0.608	16/13	0.514 [0.296–0.732]	>6.5	18.8	92.3	0.921
**TIBC [μmol/L]**	16/41	0.597 [0.432–0.761]	<52.45	56.2	70.7	0.547	14/29	0.532 [0.341–0.723]	<49.3	64.3	48.3	0.831	16/15	0.475 [0.263–0.687]	>46.95	43.8	66.7	0.921
**UIBC [μmol/L]**	16/41	0.565 [0.409–0.721]	<31.7	81.2	43.9	0.646	14/29	0.576 [0.398–0.755]	<34.4	71.4	55.2	0.608	16/15	0.475 [0.261–0.689]	>48.2	93.8	20	0.921
**CALPRO [mg/kg]**	6/14	0.571 [0.248–0.895]	<1746	33.3	100	0.700	6/7	0.690 [0.346–1]	<527.5	66.7	85.7	0.608	8/5	0.650 [0.317–0.983]	>75.5	50	80	0.763
**CRP [mg/L]**	17/42	0.541 [0.397–0.686]	<2.5	47.1	64.3	0.685	**16/29**	**0.793 [0.656–0.930]**	**<5.5**	**68.8**	**89.7**	**0.004**	17/15	0.576 [0.405–0.748]	<2.5	41.2	80	0.763

Abbreviations: Alb, albumin; AOPP, advanced oxidation protein products; AUC, area under the receiver operating curve; BILDIR, direct bilirubin; CALPRO, calprotectin; Cerulo, ceruloplasmin; CI, 95% confidence interval for area under the curve; CRP, C-reactive protein; cutoff, threshold that best separates patients in remission vs. active disease based on Youden index (the direction of the inequality (>, <) indicates the biomarker value associated with remission); GGT, gamma-glutamyl transferase; Hb, haemoglobin; IBDQ, inflammatory bowel disease questionnaire; MDA, malondialdehyde; n, total number of patients with available outcome data; N_A, number of patients with active disease included in the analysis; N_R, number of patients in remission included in the analysis; p, result of the Mann–Whitney U test comparing biomarker levels between patients with active disease and those in remission, adjusted for multiple comparisons using the Benjamini–Hochberg procedure; R-SH, plasma free thiols; SN, sensitivity; SP, specificity; SUA, serum uric acid; TAC, total antioxidant capacity; TBIL, total bilirubin; TIBC, total iron-binding capacity; UIBC, unsaturated iron-binding capacity.

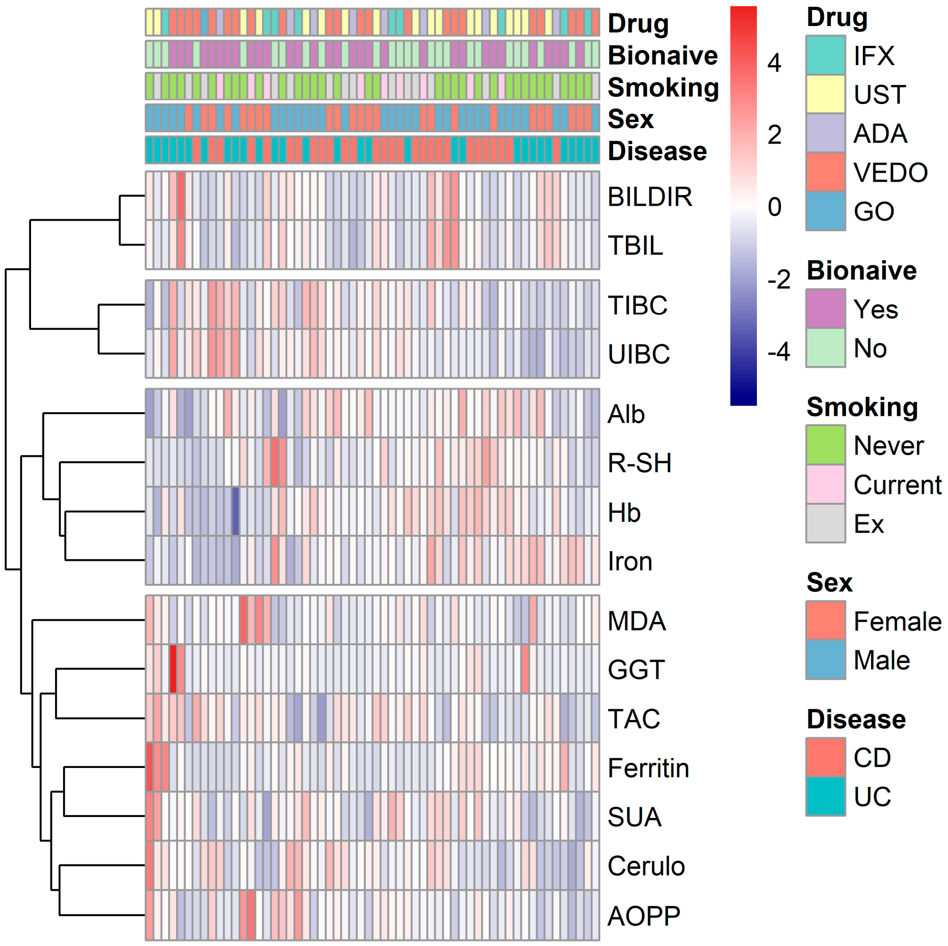

Figure S1. Heatmap showing hierarchical clustering of 15 oxidative stress-related biomarkers in patients with IBD at baseline, annotated by drug, biologic-naïve status, smoking status, sex, and IBD subtype (CD vs. UC). Patients with complete biomarker data were included (n = 58), and biomarker values were Z-score standardized. A centered color scale was applied with red indicating above-average and blue indicating below-average biomarker levels across the cohort. Rows represent individual biomarkers, and columns represent individual patients. When annotated by drug (ADA, VEDO, GO, IFX, UST), biologic-naïve status, smoking status, sex, and IBD subtype (CD vs. UC), no apparent clustering by these factors was observed. Abbreviations: Alb, albumin; ADA, adalimumab; AOPP, advanced oxidation protein products; BILDIR, bilirubin (direct); CD, Crohn's disease; Cerulo, ceruloplasmin; GGT, gamma-glutamyl transferase; GO, golimumab; Hb, hemoglobin; IFX, infliximab; IBD, inflammatory bowel disease; MDA, malondialdehyde; R-SH, plasma free thiols; SUA, serum uric acid; TAC, total antioxidant capacity; TBIL, total bilirubin; TIBC, total iron-binding capacity; UC, ulcerative colitis; UIBC, unsaturated iron-binding capacity; UST, ustekinumab; VEDO, vedolizumab.

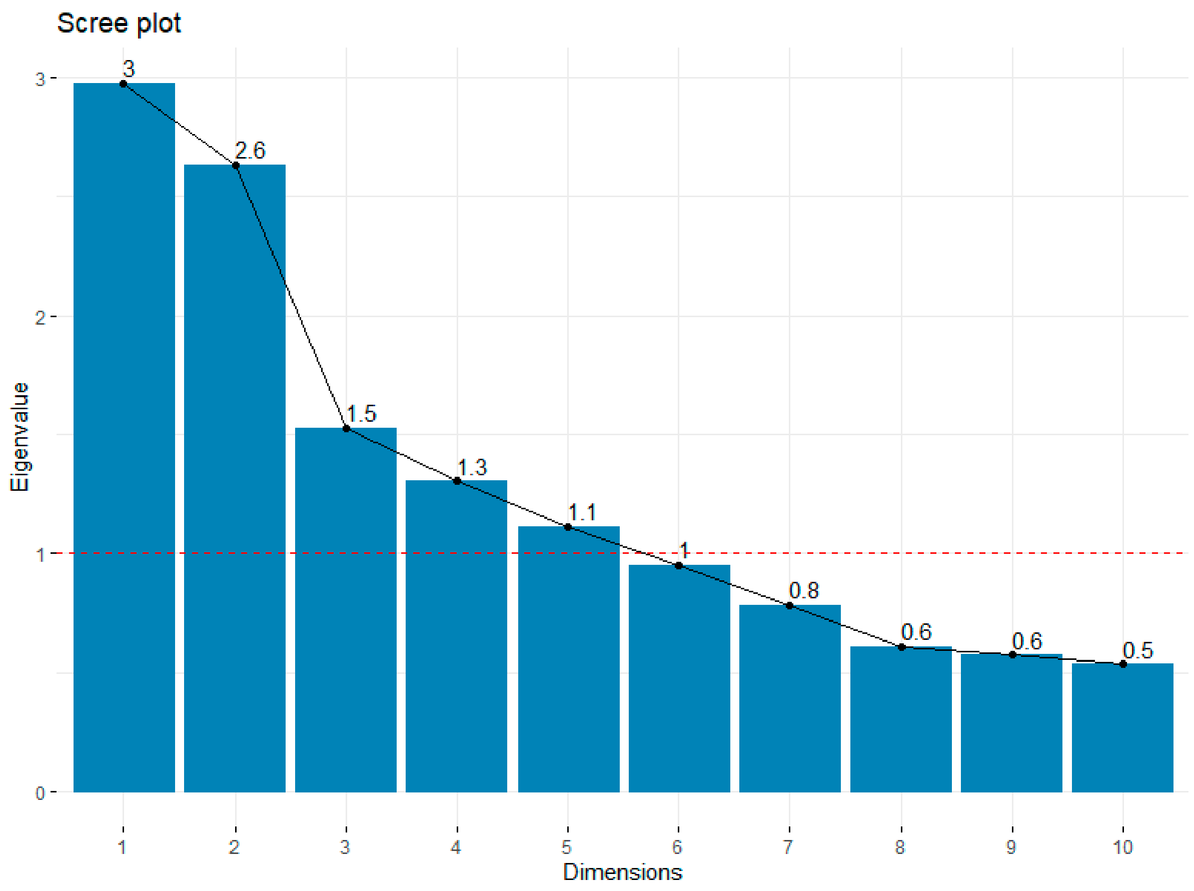

Figure S2. Scree plot showing the eigenvalues of the principal components (dimensions). The red dashed line at eigenvalue = 1 represents the Kaiser criterion, indicating that components with eigenvalues greater than 1 should be retained for further analysis.
